# Whole Exome Sequencing Study of Parkinson Disease and Related Endophenotypes in the Italian Population

**DOI:** 10.3389/fneur.2019.01362

**Published:** 2020-01-10

**Authors:** Alessandro Gialluisi, Mafalda Giovanna Reccia, Alfonsina Tirozzi, Teresa Nutile, Alessia Lombardi, Claudia De Sanctis, International Parkinson's Disease Genomic Consortium (IPDGC), Sara Varanese, Sara Pietracupa, Nicola Modugno, Antonio Simeone, Marina Ciullo, Teresa Esposito

**Affiliations:** ^1^IRCCS Neuromed, Pozzilli, Italy; ^2^Institute of Genetics and Biophysics, National Research Council, Naples, Italy

**Keywords:** Parkinson disease, genetics, whole exome sequencing, cognitive performance, motor symptoms, non-motor symptoms, subcortical volumes, polygenic scores

## Abstract

Parkinson Disease (PD) is a complex neurodegenerative disorder characterized by large genetic heterogeneity and missing heritability. Since the genetic background of PD can partly vary among ethnicities and neurological scales have been scarcely investigated in a PD setting, we performed an exploratory Whole Exome Sequencing (WES) analysis of 123 PD patients from mainland Italy, investigating scales assessing motor (UPDRS), cognitive (MoCA), and other non-motor symptoms (NMS). We performed variant prioritization, followed by targeted association testing of prioritized variants in 446 PD cases and 211 controls. Then we ran Exome-Wide Association Scans (EWAS) within sequenced PD cases (*N* = 113), testing both motor and non-motor PD endophenotypes, as well as their associations with Polygenic Risk Scores (PRS) influencing brain subcortical volumes. We identified a variant associated with PD, rs201330591 in *GTF2H2* (5q13; alternative T allele: OR [CI] = 8.16[1.08; 61.52], FDR = 0.048), which was not replicated in an independent cohort of European ancestry (1,148 PD cases, 503 controls). In the EWAS, polygenic analyses revealed statistically significant multivariable associations of amygdala- [β(SE) = −0.039(0.013); FDR = 0.039] and caudate-PRS [0.043(0.013); 0.028] with motor symptoms. All subcortical PRSs in a multivariable model notably increased the variance explained in motor (adjusted-R^2^ = 38.6%), cognitive (32.2%) and other non-motor symptoms (28.9%), compared to baseline models (~20%). Although, the small sample size warrants further replications, these findings suggest shared genetic architecture between PD symptoms and subcortical structures, and provide interesting clues on PD genetic and neuroimaging features.

## Introduction

Parkinson's Disease (PD) is one of the most common neurodegenerative disorders, affecting 1% of the population over 60 years of age, causing a progressive loss of dopaminergic neurons in the substantia nigra pars compacta (1, 2). This results in a wide phenotypic spectrum, including both motor (e.g., rigidity, tremor, and bradikynesia) and non-motor symptoms (e.g., cognitive impairment and depression) (2). PD is characterized by a complex architecture, with a number of genetic and environmental factors influencing susceptibility to the disease (3). This disorder shows an extreme genetic heterogeneity, with 10% of PD cases having Mendelian inheritance (1, 4). The genes which have been most robustly implicated in Mendelian forms of PD include *SNCA* (5), *LRRK2* (6), *PARK2* (7), *ATP13A2* (8), *PINK1* (9), *DJ-1* (10), *VPS35* (11), *DNAJC13* (12), and *GBA* (13) [see (14–16) for a review]. In these and other genes, rare mutations with both dominant (5, 6) or recessive inheritance modes (7, 9, 10) have been identified, often through genome-wide linkage studies followed by targeted genotyping [e.g., (6)] or, more recently, through Next Generation Sequencing (NGS) studies [e.g., (11, 12)]. In addition to rare mutations, also common susceptibility variants like Single Nucleotide Polymorphisms (SNPs) have been detected within these genes, e.g., in *LRRK2* and *SNCA* (16). However, the genetic variants identified so far—be they common or rare—explain only a minor part of PD heritability (17), and for a large majority of PD cases the genetic diagnosis remains unresolved. The issue of missing heritability has been tackled through different approaches in the last years, including Genome Wide Association Scans (GWAS) to identify common variants with moderate/weak effect sizes on PD susceptibility [e.g., (18)], and NGS (mostly Whole Exome Sequencing) studies to identify rare causative mutations [e.g., (3, 4, 17, 19–22)]. Moreover, the genetic architecture and the mutational spectrum of PD can vary based on the ethnic and genetic background of the population (2, 23), hence population-specific genetic studies are warranted [as in (17, 21)].

Large-scale genomic studies carried out so far have scarcely investigated inter-individual variation in PD endophenotypes like neurological scales (3, 4, 17–22, 24). A GWAS study of age-at-onset in 25,568 PD cases reported two genome-wide significant associations within *SNCA* and *TMEM175* (25), while other preliminary GWAS of cognitive performance and motor symptoms progression are ongoing (26, 27). Other SNP-based genomic studies tested associations of Polygenic Risk Scores (PRS) for PD with alpha-synuclein levels in the cerebrospinal fluid, age-at-onset of the disease, motor/cognitive symptoms and PD status [as reviewed in (28)], detecting significant associations with PD risk (29), earlier PD onset (29, 30), and faster motor and cognitive decline (31). In addition, the largest case-control GWAS on PD carried out so far—involving the analysis of ~56,000 cases and 1.4 million controls—identified significant genetic correlations with structural neuroimaging measures like intracranial and putamen volume (24). However, PRS analyses of Parkinson neuroimaging correlates were never reported. Overall, no NGS study so far focused on identifying genetic variants associated with PD endophenotypes, and there is a paucity of genomic studies doing so, in particular with motor, cognitive and non-motor scales, as well as with neuroimaging traits related to PD risk and symptoms, like subcortical volumes (32–35).

Here, we present the first Whole Exome Sequencing (WES) analysis investigating continuous PD endophenotypes, and PD genetic susceptibility in mainland Italy. Through an exploratory multi-stage approach, we first performed rare variant prioritization and case-control association testing, attempting replication of findings in an international cohort of PD cases and controls of European ancestry (22). Then, we carried out Exome-Wide Association Scans with continuous neurological scales related to PD, assessing both motor and non-motor symptoms, to identify common variants potentially affecting these domains. Finally, we performed PRS analyses to test associations between polygenic scores influencing subcortical volumes and the above mentioned scales. Our study provides a contribution to the research on the genetic basis of PD, focusing on motor, non-motor, and neuroimaging measures related to the disease.

## Subjects and Methods

### PD Cohorts

Inclusion criteria for the participants to the study were reported Italian ancestry and a clinical diagnosis of PD by a qualified neurologist, according to published diagnostic criteria (see Supplementary Methods and (36).

Four hundred and seventy-two PD patients [288 males; 196 familiar cases; mean (SD) age of 66.6 (8.8) years] were recruited at the Parkinson Center of the specialized clinics IRCCS Neuromed, Pozzilli, Italy, between June 2015 and December 2017. They underwent a detailed phenotypic assessment and diagnostic protocol, which included neurological examination and evaluation of non-motor domains (see Supplementary Methods for details). The mean (SD) age at diagnosis was 58.3 (10.0) years. Along with patients, 121 non-consanguineous family members with no neurological signs or symptoms of PD at the time of recruitment were involved in the study, by donating blood samples for targeted genetic analyses [mean (SD) age 62.9 (9.1) years; 44 males].

An additional cohort from mainland Italy was involved in the study, recruited at the Parkinson Institute of Istituti Clinici di Perfezionamento in Milan (hereafter called ICP). This included 82 related FPD patients of Italian ancestry, coming from 42 families with two or more first-degree relatives affected by PD [mean age 66.7 (10.4) years; mean (SD) age at diagnosis 60.69 (10.62) years; 41 males]. Further details on these cohorts are reported in Table S1a.

The project was approved by the ethical committees of IRCCS Neuromed, Pozzilli, and of ICP, Milan, and written informed consent was obtained from all the participating subjects.

### Whole Exome Sequencing, Quality Control (QC) and Annotation

162 PD cases, including 90 familiar cases (FPD, 42 from Neuromed and 48 from ICP) and 72 sporadic cases (SPD, from Neuromed), underwent WES analysis (see Table S1b for details) through the Illumina® HiSeq2000 platform (Illumina, San Diego, CA, USA), using the SureSelect All Exome kit v6 (Agilent® Technologies, Santa Clara, CA, USA) for enrichment of exonic regions. The alignments of reads to GRCh37/hg19 was performed using the Burrows Wheeler Aligner (BWA) MEM v0.7.5 (37). After removal of duplicate reads through Picard MarkDuplicates command, single nucleotide variants (SNVs) and insertions/deletions (indels) were called using HaplotypeCaller and GenotypeGVCFs in Genome Analysis Toolkit (GATK) v3.5-0-g36282e4 (38). Variant calls with total depth (DP) <8 and genotype quality (GQ) <50 were set to missing, and variants with Minor Allele Count (MAC) = 0, number of alternative alleles ≠ 2 and call rate <95% were filtered out, as well as samples with identical-by-descent (IBD) sharing and sex mismatches, and samples with call rate <90% and intraspecific contamination rate >7%. Similarly, samples were checked for absence of outliers in terms of genome-wide homozygosity, number of singleton variants, and genetic ancestry [through Multidimensional Scaling Analysis in PLINK v 1.9; (39)]. 123 PD cases (52 FPD + 71 SPD) and 334,671 variants (321,967 SNPs + 12,704 indels) passed QC. These variants were annotated to genes (within 10 kb from transcription start/stop site) through Annovar version 1-2-2016 (40) and Ensembl Variant Effect Predictor (VEP) v88 (41). Further details on genotype calling and QC are reported in Supplementary Methods.

### Variants Prioritization, Validation, and Genetic Association Analysis With PD Status

**Figure 1:**
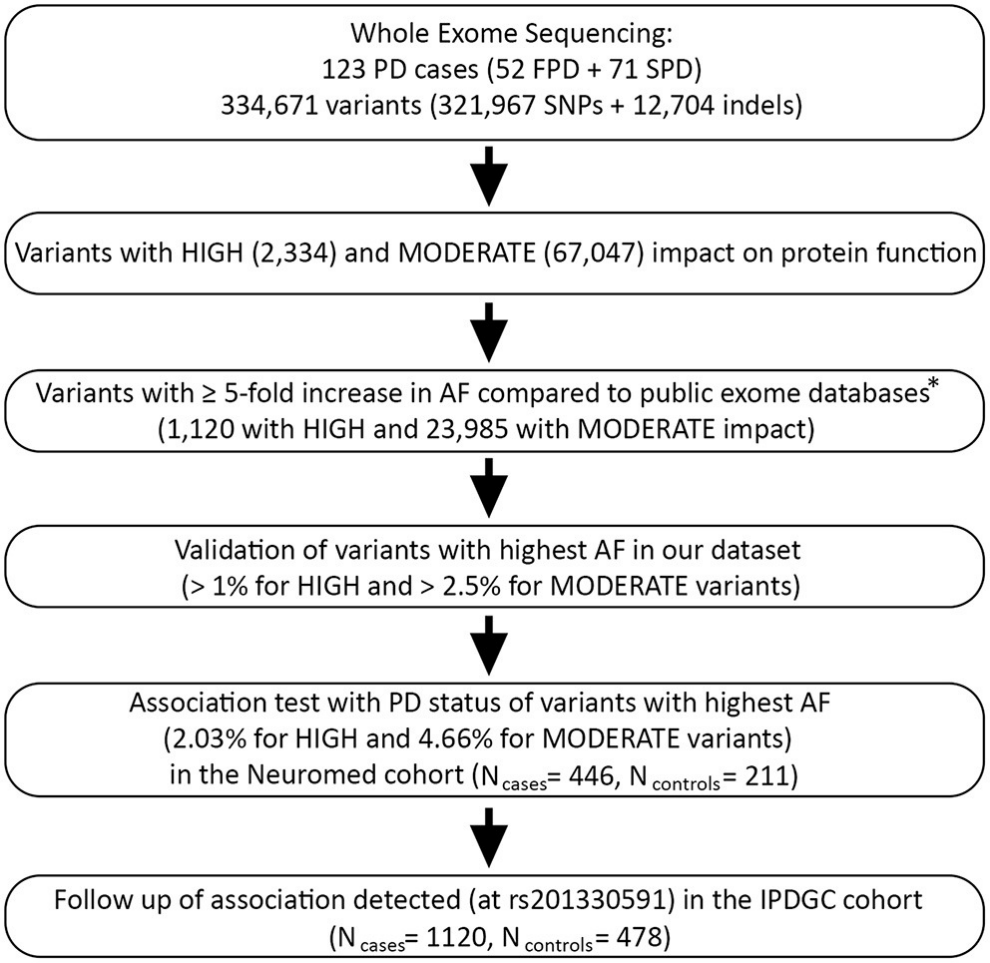
Bioinformatic pipeline applied in the present study for PD causative variants prioritization and case-control association testing. PD, Parkinson disease; FPD/SPD, familial/sporadic Parkinson disease; IPDGC, International Parkinson Disease Genetics Consortium (22). *Public exomes databases: 1000G EUR, European Sample of the 1000 Genomes project, phase 3 (*N* = 503) (42); ESP EA, NHLBI Exome Sequencing Project 6500 release si-v2, European ancestry (*N* = 4,300) (43); ExAC NFE, Exome Aggregation Consortium version 0.3.1, Non-Finnish Europeans (*N* = 33,370) (44).

Among 334,671 variants passing QC, we attempted to detect rare variants potentially associated with PD status in our dataset (123 PD cases). To this purpose, we applied the following bioinformatic pipeline (resumed in Figure 1):

1. We selected variants with predicted high or moderate impact on protein function, based on VEP annotation (41). These included 2,334 variants assumed to have high (disruptive) impact on the protein, probably causing protein truncation, loss of function or triggering nonsense mediated decay (hereafter called HIGH variants), and 67,047 non-disruptive variants that might change protein effectiveness (hereafter called MODERATE variants; see Table S2 for a detailed classification).
2. We retained variants with an alternative allele frequency (AF) at least five times higher than in three WES databases representative of the European population, namely 1,000 Genomes EUR (European Samples of the 1,000 Genomes project, phase 3 v5; *N* = 503) (42), ESP EA (European American samples of NHLBI Exome Sequencing Project 6500 release si-v2; *N* = 4,300) (43), and ExAC NFE (Non-Finnish Europeans of the Exome Aggregation Consortium version 0.3.1; *N* = 33,370) (44). This resulted in the selection of 1,120 HIGH and 23,985 MODERATE variants.
3. We ranked resulting variants based on decreasing AF, and validated top-ranked variants in our dataset—namely HIGH variants with AF > 1% and MODERATE variants with AF > 2.5%—through Sanger sequencing or PCR (see Supplementary Methods; Tables S3a,b).
4. We performed targeted genotyping and case-control association analysis of the most frequent validated variants within each of the two functional annotation classes, namely HIGH and MODERATE impact variants. More specifically, we tested two HIGH variants (AF = 2.03%) and one MODERATE variant (AF = 4.66%) in our cohort (Table S4). This analysis was performed on 446 PD cases and 211 controls, which included 121 non-consanguineous relatives of PD patients and 90 unscreened controls (pseudo-controls) belonging to the general Italian population. Association analysis was performed through an allelic Fisher Exact Test with adaptive permutations in PLINK (see Supplementary Methods). Since age and sex were missing for pseudo-controls, no covariates were used in this analysis to avoid a substantial loss of sample size.
5. We attempted a replication of a significant association observed (rs201330591), in an independent case-control WES study of 1,148 young-onset unrelated PD cases (average age at onset 40.6 years; range 35–56 years) and 503 control participants of European ancestry (IPDGC cohort) (22). As above, we performed an allelic Fisher Exact Test and then meta-analyzed the resulting association with that observed in the Italian cohort, through a Mantel-Haenszel meta-analysis in R (45) (see Supplementary Methods for further details).

### Exome-Wide Association Study With PD Endophenotypes

We tested common variants detected through WES for association with three continuous scales which assessed different domains usually affected in PD (Tables S5a,b). These scales included the Movement Disorder Society revised version of the Unified Parkinson's Disease Rating Scale Part III (hereafter called UPDRS) (46), which assessed motor symptoms; the Montreal Cognitive Assessment (MoCA) (47), which measures general cognitive abilities; and a modified version of Non-Motor Symptoms Scale for Parkinson Disease (hereafter called NMS) (48), which tests non-motor symptoms (see Supplementary Methods for details). Indeed, these scales represent useful endophenotypes which allow to disentangle the genetic basis of PD at a fine-grained resolution, as done elsewhere (49–51).

After genotypic and phenotypic QC (described in Supplementary Methods), 110,803 common autosomal variants (MAF > 5%) in 113 PD cases were available for association testing, which was carried out in two steps. First, we performed univariate linear mixed effect models in EMMAX [version March 2010; (52)], to identify genetic effects on each single neurological scale tested. Then, in light of the moderate correlations among these scales (Table S5b), we carried out a multivariate genetic association analysis on all the three scales together, through TATES (53), to identify relational pleiotropic effects on the domains assessed. These analyses were adjusted for different covariates, including PD familiarity, sex, age, pharmacological treatment status (ON/OFF), years of disease, daily L-Dopa dosage, and 10 genetic ancestry components. The significance threshold of the multivariate analysis was corrected for the number of LD-independent SNPs tested (α = 0.05/56,588 = 8.84 × 10^−7^), as computed by the Genetic Type I error calculator (GEC) (54), while for univariate tests we applied an additional Bonferroni correction for the number of scales tested (α = 2.95 × 10^−7^).

To follow-up on the results of the exome-wide association study (EWAS), we performed a targeted genotyping and association testing of the top hit identified in the whole Neuromed cohort (472 PD cases), through linear regression models with adaptive permutations in PLINK, using the same covariates as above except for genetic ancestry (see Supplementary Methods). After single univariate association tests with PD endophenotypes, we then combined the results into a multivariate association analysis through TATES.

### Polygenic Risk Score (PRS) Analyses of PD Endophenotypes

We used exome-wide genetic data to build Polygenic Risk Scores (PRSs) influencing brain subcortical volumes, which were trained using summary statistics of a previous large independent GWAS (N_max_ = 30,717) (55). This analysis was motivated by previous literature reporting both structural and functional alterations of several subcortical structures in PD (32–35), for which these are often considered useful neuroimaging correlates. First we computed standardized best-fit polygenic scores through PRSice-2 (56), over varying association significance thresholds in the training GWAS (ranging from 5 × 10^−8^ to 1), for nucleus accumbens, caudate, putamen, pallidum, amygdala, hippocampus and thalamus volume. Then we tested the resulting scores for association with UPDRS, MoCA and NMS scales through generalized linear models [*glm()* function in R], in a random extraction of one individual per family in our cohort (four relatives removed). These models were adjusted for PD familiarity, sex, age, pharmacological treatment status (ON/OFF), years of disease, daily L-Dopa dosage, and 10 genetic ancestry components, as above. Since these subcortical structures are thought to be functionally connected in complex connectivity networks and the pattern of atrophy is often spread across these networks in PD pathology (32, 57), we performed multivariable models, testing all the PRSs built simultaneously, for each PD endophenotype. First, we assessed multivariate associations of each subcortical PRS to detect evidence at the specific structure level, applying a Benjamini-Hochberg correction for three different PD scales and five independent latent subcortical traits tested (58), as revealed by Matrix Spectral Decomposition applied to the phenotypic correlation matrix of these measures (59). Then, to have a measure of the total variance of PD endophenotypes explained by the polygenic scores, we compared adjusted Nagelke's R^2^ values of the multivariable models including all the subcortical PRSs and covariates (hereafter called full PRS models) with the baseline models including only covariates (see above).

## Results

Exome-wide, we prioritized three validated variants showing the highest alternative allele frequency among those with high and moderate impact on protein function, in our cohort of patients. These included rs772162369 in *MFSD6L* and rs56407180 in *KALRN* among HIGH variants (AF = 2.03%), and rs201330591 in *GTF2H2* among MODERATE variants (AF = 4.66%) (Table S4). These variants were further tested for association with PD in the whole Neuromed cohort, which revealed a significant associations with PD for rs201330591 [uc011crt.2:exon7:c.T217A:p.S73T; alternative T allele: OR [CI] = 8.16 [1.08; 61.52], *p* = 0.02, FDR < 0.05; see Table 1]. However, this association was not replicated in the IPDGC cohort [rs201330591: OR [CI] = 1.12 [0.41; 3.04], *p* = 0.83], nor in the following meta-analysis with our study (see Table S6).

**Table 1:** Association statistics (OR and 95% Confidence Interval) of the most frequent genetic variants detected through the variant prioritization pipeline in our PD callset.

**Variant (dbSNP147)**	**Chr**	**Bp**	**REF**	**ALT**	**AF_Cases**	**AF_Ctrls**	**OR [CI]**	**P**	**FDR**	**Gene**	**Location (consequence)**	**VEP impact**
rs772162369	17	8701167	CA	C	0.0179	0.0024	7.58 [1.00; 57.35]	0.061	0.092	MFSD6L	Exonic (frameshift deletion)	H
rs56407180	3	124303696	C	T	0.0079	0.0048	1.66 [0.34; 8.01]	0.786	0.786	KALRN	Exonic (stop gain)	H
**rs201330591**	**5**	**70351261**	**A**	**T**	**0.0191**	**0.0024**	**8.16** **[1.08; 61.52]**	**0.016**	**0.048**	**GTF2H2**	**Exonic (missense)**	**M**

*Variants which showed statistically significant associations (FDR < 0.05) are highlighted in bold*.

*REF/ALT, reference/alternative allele; AF_Cases/AF_Ctrls, alternative allele frequency in cases/controls; OR [CI], Odds Ratio referring to ALT allele (95% Confidence Interval); P, empirical p-value based on allelic Fisher Exact Test with adaptive permutations in PLINK; FDR, Benjamini-Hochberg False Discovery Rate adjusted p-value*.

Exome-wide association analyses with different scales assessing motor and non-motor PD symptoms revealed no significant genetic associations surviving Bonferroni correction, neither in a univariate (Table 2; Figures S1a–f), nor in a multivariate setting (Table S7; Figures S1g,h). The most significant effects were observed for rs3835072 [GAA/G, MAF ~ 40%; *p* = 6.69 × 10^−7^, β (SE) = 0.089 (0.017) for major allele GAA], an intronic indel located in the *CCT7* gene. Although this effect met nominal exome-wide significance (α = 8.84 × 10^−7^), it did not survive correction for testing of multiple scales (α = 2.95 × 10^−7^). Other close variants showed comparable associations and were all in high LD (r^2^ > 0.75; see Table 2), suggesting they tagged the same genetic effect. Similarly, rs3835072 showed the most significant multivariate association with the different PD endophenotypes tested (*p* = 1.88 × 10^−6^), supported by a nominally significant effect on UPDRS (*p* = 0.033, β (SE) = −0.038 (0.017)], in addition to the one detected with MoCA. Again, this variant approached but did not met exome-wide significance (α = 8.84 × 10^−7^; Table S7). A follow-up association test in the whole Neuromed cohort revealed only a trend of association of this variant with MoCA [*p* = 0.067; β (SE) = 0.018 (0.010) for major allele GAA], while no significant association was observed neither with univariate UPDRS/NMS scales, nor in a multivariate setting (*p* = 0.19; Table S8).

**Table 2:** Most significant single variant associations (*p* < 10^−5^) detected in the univariate EWAS of three continuous scales assessing PD endophenotypes (see abbreviations below).

**SNP**	**Chr**	**Bp**	**A1**	**A2**	**A1 Freq (%)**	**β (A1)^a^**	**SE^a^**	**P^a^**	**Gene (within 10 kb)**	**Position (distance in kb)**	**Functional implication**	**LD (R^**2**^) with local top hit**	**Trait**
rs3835072	2	73467676	GAA	G	60.2	0.090	0.017	6.7 × 10^−7^	CCT7, PRADC1	Intronic, upstream (7)	Splicing	NA	MoCA
rs1864492	2	73429808	G	C	71.9	0.094	0.019	3.0 × 10^−6^	NOTO	Exonic	Missense	0.52	MoCA
rs1053329	2	73461483	C	A	65.9	0.084	0.017	3.1 × 10^−6^	CCT7, PRADC1, SMYD5	Exonic (5'-UTR), upstream (1), downstream (7)		0.81	MoCA
rs2288631	2	73455747	C	T	65.9	0.084	0.017	3.1 × 10^−6^	PRADC1, SMYD5, CCT7	Intronic, downstream (1), upstream (6)		0.81	MoCA
rs7851	2	73478461	A	T	65.9	0.084	0.017	3.1 × 10^−6^	CCT7, FBXO41	Exonic, downstream (3)	Synonymous	0.81	MoCA
rs909065	2	73477351	T	C	65.9	0.084	0.017	3.1 × 10^−6^	CCT7, FBXO41	Intronic, downstream (4)		0.81	MoCA
rs2303904	2	73447775	A	G	58	0.085	0.017	3.7 × 10^−6^	SMYD5, PRADC1, NOTO	Intronic, downstream (7), downstream (9)		0.92	MoCA
rs144206161	19	3453692	G	A	94.7	0.188	0.038	4.6 × 10^−6^	NFIC	Intronic		NA	MoCA
rs2652189	5	174935860	T	G	84.9	0.113	0.024	6.7 × 10^−6^	SFXN1	Intronic		NA	UPDRS
rs17127600	14	92279983	T	G	85.4	−0.083	0.017	7.0 × 10^−6^	TC2N	Intronic		NA	NMS
rs2237051	4	110901198	G	A	57.5	0.078	0.016	7.5 × 10^−6^	EGF	Exonic	Missense	NA	UPDRS
rs17008885	2	73450139	A	T	64.6	0.082	0.017	8.9 × 10^−6^	SMYD5, PRADC1	Intronic, downstream (5)		0.76	MoCA

a *Univariate association p-values as computed by EMMAX linear mixed model are reported, along with beta (β) values referring to major allele (A1) and relevant Standard Errors (SE)*.

*UPDRS, Movement Disorder Society revised version of the Unified Parkinson's Disease Rating Scale—Part III (46); MoCA, Montreal Cognitive Assessment (47); NMS, modified version of the Non-Motor Symptoms Scale for Parkinson Disease (see Supplementary Methods) (48)*.

Multivariable associations analyses with standardized subcortical polygenic scores revealed statistically significant associations of UPDRS score with amygdala- [β (SE) = −0.039 (0.013), *p* = 0.004, FDR = 0.039] and caudate-PRS [β (SE) = 0.043 (0.013), *p* = 0.001, FDR = 0.028]. Full results of the multivariable models for the three PD scales tested are reported in Table 3. Overall, the multivariable association model including all the subcortical PRSs (full PRS model) explained 38.6% of variance in UPDRS scores, vs. 20.3% in the baseline model (including only covariates). A smaller discrepancy was observed for the other PD endophenotypes, where full PRS models explained 32.2 and 28.9% of the total variance in MoCA and NMS scores (vs. 20.3 and 20.7% in the baseline models), respectively.

**Table 3:** Results of multivariable regression models testing associations of PD endophenotypes with best-fit Polygenic Risk Scores (PRSs) for subcortical volumes in the Neuromed cohort.

**PRS**	**UPDRS**		**MoCA**		**NMS**	
	**Beta (SE)**	***p* (FDR)**	**Beta (SE)**	***p* (FDR)**	**Beta (SE)**	***p* (FDR)**
Accumbens	−0.038 (0.015)	0.012 (0.082)	−0.006 (0.013)	0.682 (1)	0.006 (0.010)	0.572 (1)
**Amygdala**	**−0.039** **(0.013)**	**0.004** **(0.039)**	−0.03 (0.016)	0.055 (0.482)	0.027 (0.012)	0.027 (0.576)
**Caudate**	**0.043** **(0.013)**	**0.001** **(0.028)**	−0.025 (0.013)	0.065 (0.482)	0.015 (0.010)	0.134 (0.705)
Hippocampus	0.016 (0.010)	0.128 (0.537)	−0.015 (0.013)	0.243 (0.851)	0.009 (0.006)	0.098 (0.684)
Pallidum	0.009 (0.013)	0.483 (1)	−0.018 (0.010)	0.069 (0.482)	0.004 (0.011)	0.745 (1)
Putamen	−0.033 (0.015)	0.034 (0.177)	−0.024 (0.015)	0.115 (0.482)	0.009 (0.008)	0.287 (1)
Thalamus	−0.002 (0.010)	0.853 (1)	0.021 (0.012)	0.100 (0.482)	0.013 (0.007)	0.090 (0.684)

*Here we report Betas (β) and relevant Standard Errors (SE) of the multivariable regression performed in a random extraction of one individual per family, along with association p-values and relevant (Benjamini-Hochberg) False Discovery Rates. Statistically significant associations (FDR < 0.05) are highlighted in bold*.

*UPDRS, Movement Disorder Society revised version of the Unified Parkinson's Disease Rating Scale—Part III (46); MoCA, Montreal Cognitive Assessment (47); NMS, modified version of the Non-Motor Symptoms Scale for Parkinson Disease (see Supplementary Methods) (48)*.

## Discussion

In this paper, we report an exploratory WES analysis of 123 PD cases from Italy. Although a previous study analyzed PD cases from an Italian genetic isolate, Sardinia (17), this represents the first WES study focused on PD patients from mainland Italy, the largest ever carried out in the country, and the richest in terms of phenotypes assessed. Indeed, in spite of the relatively small sample size sequenced and of the availability of exome (rather than whole genome) data, which represent the main limitations of the present study, we exploited the wealth of neurological scales assessed to carry out an exome-wide association study of motor and non-motor PD endophenotypes. Moreover, we tested associations of the scales available—namely UPDRS, MoCA and NMS—with PRSs known to influence subcortical volumes, which have long been considered as neuroimaging correlates of PD and neurodegeneration (32–35). To our knowledge, this study represents the first attempt to test genetic associations with neurological scales in PD at the exome-wide level.

Through a stepwise approach, we identified a genetic variant with a frequency notably higher than in published WES databases and a significant association with PD in an extended analysis of our cohort, namely rs201330591, encoding a Serine-to-Threonine change in *GTF2H2* (*General Transcription Factor IIH Subunit 2*; 5q13). *GTF2H2* has been previously implicated as a modifier gene in spinal muscular atrophy (SMA), an autosomal recessive neurodegenerative disorder characterized by progressive death of motor neurons, implying proximal muscle weakness, and wasting in the absence of sensory signs. This gene is located not far from the causative gene of SMA, *SMN1* (*Survival Motor Neuron 1*), and deletions involving this gene have been detected in severe forms of the disease (60). It encodes a subunit of the TFIIH transcription factor, which has been also implicated in Cockayne syndrome, a rare disease characterized by progeria and nervous system abnormalities, among other signs (61). However, the association detected was not replicated in the IPDGC cohort (22), which suggests caution in the interpretation of this finding. This may be due to several reasons, including different recruitment and filtering criteria of the two studies, the extreme genetic heterogeneity of PD, the lack of power in our analyses or the possibility that false positives were detected in the discovery cohort, due to its small sample size. Further replication attempts are warranted to support this finding.

The exome-wide analysis of continuous PD endophenotypes revealed an association approaching exome-wide significance at rs3835072, both in a univariate setting with MoCA score (representing general cognitive performance) and at the multivariate level, including other motor (UPDRS) and non-motor PD endophenotypes (NMS). rs3835072 is an intronic indel predicted to alter splicing in the *CCT7* gene *(chaperonin containing TCP1, subunit 7*; 2p13.2). This gene encodes a member of the chaperonin containing TCP1 (CCT) complex, which is impaired in severe neuropathies and in neurodegenerative disorders like Alzheimer's Disease (AD), where it is thought to promote toxic protein aggregates and cell death (62). Interestingly, the leading association signal identified at rs3835072 was with cognitive performance, which is impaired both in AD and in PD (63). However, this association only approached significance in an extended follow-up analysis of our PD cohort, which does not support a significant influence of this gene on cognitive performance.

The most interesting findings of the present work come from associations analyses of polygenic risk scores (PRSs) influencing brain subcortical volumes with the continuous PD endophenotypes available. Multivariable regression models analyzing all the subcortical polygenic scores together (full PRS models) revealed a notable increase in the proportion of variance explained for the PD scales tested, compared to the baseline models including only covariates (see Methods section). In particular, the fraction of variance explained almost doubled for motor symptoms (UPDRS), increasing from ~20% in the baseline model to ~39% in the full model. For non-motor symptoms scales (MoCA and NMS), the increase in variance explained by the full PRS model was less sharp (32 and 29%, respectively), but still evident. This suggests that the genetic underpinnings of brain subcortical structures may be important in influencing PD symptoms, especially for the motor domain. Among the seven subcortical PRS tested in the multivariable model, we observed significant associations of amygdala- and caudate-PRS with UPDRS. These findings do not support the significant bivariate genetic correlation between putamen volume and PD risk recently reported (24), a discrepancy which may be explained by the different methodologies used to investigate genetic overlap and by the low power provided by our study. Moreover, while the inverse association observed between the amygdala-PRS and motor symptoms is in line with its reported atrophy in PD (64, 65), the positive association observed for the caudate polygenic score is in contrast with previous neuroimaging observations of reduced caudate volumes in Parkinson patients (66, 67), although these associations are often localized and not always consistent (33, 67). A potential explanation for this discrepancy may be again type I error, due to the small sample size of our study. Alternatively, since caudate hypertrophy has been associated with vascular parkinsonism (68) and compensatory hypertrophy mechanisms have been reported for some subcortical structures in PD (64, 66), we may hypothesize that some PD patients may have a genetic predisposition to atrophy/hypertrophy in different subcortical structures, each representing a unique “mosaic” in terms of liability to motor and non-motor neurological symptoms. Although we are still far from a comprehensive view of structural brain changes in PD, multi-omic studies involving neuroimaging, clinical and genetic levels may help to verify this hypothesis.

Overall, the evidence reported here suggests that it is likely low power the current bottleneck in the research on the genetic bases of such a heterogeneous disorder like PD (20), and underlines the need of collaborative efforts to homogenize genetic analyses and increase sample size in WES studies of the disease. In addition, studies exploiting diverse phenotypic, pharmacological and clinical information can provide clues into the neurobiological basis of the disease. Overall, this paper represents an exploratory attempt in this sense, providing interesting insights into the shared genetic bases of PD symptoms and brain subcortical structures, in spite of the small sample size. This suggests further collaborative investigations in order to elucidate the genetic underpinnings of Parkinson Disease, its neurological endophenotypes and neuroimaging correlates.

## Data Availability Statement

Raw WES data which were analyzed in the present manuscript will be made available upon request to the corresponding author, in a way which does not affect privacy of the patients involved in the present study.

## URLs

Annovar: http://annovar.openbioinformatics.org/en/latest/
Variant Effect Predictor (VEP): https://www.ensembl.org/info/docs/tools/vep/index.html
Genome Analysis Toolkit (GATK): https://software.broadinstitute.org/gatk/
Burrows Wheeler Aligner (BWA): http://bio-bwa.sourceforge.net/
Samtools: http://samtools.sourceforge.net/
Picard: http://broadinstitute.github.io/picard
Vcftools: https://vcftools.github.io/index.html
PLINK: https://www.cog-genomics.org/plink/1.9/
1000 Genomes Project: ftp://ftp.1000genomes.ebi.ac.uk/vol1/ftp/
NHLBI Exome Sequencing Project: https://evs.gs.washington.edu/EVS/
Exome Aggregation Consortium: http://exac.broadinstitute.org/
Rmeta package: https://cran.r-project.org/web/packages/rmeta/index.html
EMMAX: http://genetics.cs.ucla.edu/emmax/index.html
TATES: https://ctg.cncr.nl/software/tates
GEC: http://grass.cgs.hku.hk/gec/
Human Integrated Protein Expression Database: http://www.genecards.org/.

## Ethics Statement

The studies involving human participants were reviewed and approved by IRCCS Neuromed. The patients/participants provided their written informed consent to participate in this study.

## Author Contributions

TE, AS, and MC designed and supervised the study. NM, SV, SP, and MR recruited the patients, carried out phenotypic assessment, and collected the data. MR and AT carried out database curation, and performed sample management and bio-banking, along with AL and CD. AG and TN performed quality control and analysis of WES and other genotype data, under the supervision of MC. The International Parkinson's Disease Genomic Consortium (IPDGC) provided the replication data set and relevant statistics. MR and AT performed wet lab experiments, under the supervision of TE. AG wrote the manuscript, with contributions and final approval by all the co-authors.

### Conflict of Interest

The authors declare that the research was conducted in the absence of any commercial or financial relationships that could be construed as a potential conflict of interest.
