# Supplementary material for: Whole Exome Sequencing Study of Parkinson Disease and Related Endophenotypes in the Italian Population
Source: Front Neurol. 2020 Jan 10;10:1362. doi: 10.3389/fneur.2019.01362 (PMC6965311; doi:10.3389/fneur.2019.01362)
Supplement: Supplementary file 1 [file Table_3.DOCX]

**Supplementary Methods**

*Phenotypic Assessment of PD cases*

All the cases involved in the study were diagnosed with PD by a qualified neurologist, according to published diagnostic criteria, which included rigidity, postural instability, resting tremor and positive response to levodopa treatment (1). Where diagnosis was uncertain, dopaminergic loss observed through neuroimaging techniques (PETscan or DaTscan) was used to confirm PD diagnosis. PD patients underwent a detailed phenotypic assessment, which included neurological examination and evaluation of non-motor domains (see below). Information about family history, demographic characteristics, anamnesis, and pharmacological therapy was also collected.

The Movement Disorder Society revised version of the Unified Parkinson’s Disease Rating Scale Part III (18 items, maximum score 72; hereafter called UPDRS) (2) was used to assess clinical motor symptoms. These included language, facial expressions, tremor, rigidity, agility in movements, stability, gait and bradykinesia. Cognitive abilities were tested through an Italian validated version of the Montreal Cognitive Assessment (MoCA) (3). Cognitive domains assessed include short-term memory (5 points); visuospatial abilities via clock drawing (3 points), and a cube copy task (1 point); executive functioning via an adaptation of Trail Making Test Part B (1 point), phonemic fluency (1 point), and verbal abstraction (2 points); attention, concentration, and working memory via target detection (1 point), serial subtraction (2 points), digits forward and backward (1 point each); language via confrontation naming with low-familiarity animals (3 points), and repetition of complex sentences (2 points); and orientation to time and place (6 points). The total score was given by the sum of these domains, then divided by the maximum score which could be obtained (30). Where one or more domains could not be tested (e.g. visuospatial tasks, due to unavailability of optical devices), the corresponding score was subtracted from the maximum total score. Non motor symptoms were assessed through an Italian validated version of Non Motor Symptoms Scale (NMS) for Parkinson Disease (4). This scale tests 9 items, including cardiovascular domain, sleep/fatigue, mood/cognition, perceptual problems/hallucinations, attention/memory, gastrointestinal, urinary, sexual function, and ability to taste or smell. For each item, both severity and frequency of symptoms is measured, so that the scale accounts for both aspects. This scale is available in (4) (esupp. File 1). Here, the sleep domain was slightly modified by adding a further question on the occurrence of vivid dreams. This question was treated as all the others, i.e. the severity of impairment was scored from 0 (no symptoms) to 3 (severe impairment), and the frequency of impairment was scored from 0 (less than once a week) to 4 (daily impairment), then the total score of the sub-item was computed as the product of severity by frequency, and added to the scores of the other sub-items. For this reason, and due to the high missing rate of sub-items in the sexual domain, we computed the NMSS total score as the sum of all the items which were answered, divided by the maximum total score which could be obtained. This produced a continuous score ranging between 0 and 1 (hereafter called NMS).

*Whole Exome Sequencing (WES): protocol and quality control*

162 PD cases, including 90 familiar cases (FPD, 42 from Neuromed and 48 from ICP) and 72 sporadic cases (SPD, from Neuromed), underwent Whole Exome Sequencing (WES), which was carried out at Helmotz Zentrum, Munich, Germany. Genomic DNA was isolated from peripheral blood lymphocytes by Blood and Cell Culture DNA Midi Kit (QIAGEN, Hilden, Germany). Exonic regions were enriched using the SureSelect All Exome kit v6 (Agilent® Technologies, Santa Clara, CA, USA) based on DNA fragmentation and capture. Exomes were barcoded and sequenced using the Illumina® HiSeq2000 platform (Illumina, San Diego, CA, USA). Average exome coverage was 148x and at least 20x for 98.9% of the target, with transition-to-transvertion (Ts/Tv) ratio of 3.14.

After removal of samples with high intraspecific contamination rate (> 7%), raw Whole Exome Sequencing data underwent the following genotype calling and quality control (QC) pipeline, using Burrows Wheeler Aligner (BWA) v0.7.5 (5), Genome Analysis Toolkit (GATK) v3.5-0-g36282e4 and Picard software (6). More specifically, we followed the GATK best practice guidelines (available at <https://software.broadinstitute.org/gatk/best-practices/>), as described below:

1. alignment of the 100-bp paired-end reads to the GRCh37/hg19 human reference genome through bwa-mem command, then merging with samtools ;
2. removal of duplicate reads through Picard MarkDuplicates (standard options);
3. genotype calling per sample through HaplotypeCaller (BP_RESOULTION option) and GenotypeGVCFs in GATK.
4. joint genotyping of samples through GenotypeGVCFs commands in GATK;
5. Variant Quality Score Recalibration (VQSR) of genotypes, which in turn consisted of i) building a gaussian mixture model based on several quality parameters of variants from specific training sets (VariantRecalibrator) and then recomputing the quality score of the variants called in our callset, based on such models (ApplyRecalibration). VQSR was conducted separately on SNPs and Indels, using a truth sensitivity threshold of 99.0 for SNPs and 90.0 for indels, respectively.
6. variant calls with total depth (DP) < 8 and genotype quality (GQ) < 50 were set to missing (in vcftools);
7. genotype posterior probability of variants was then computed using 1000G phase 3 v4 sites as supporting callset (CalculateGenotypePosteriors in GATK).

Then, we performed additional QC steps to ensure high quality of genetic data, through dedicated software vcftools v0.1.12b (7) and PLINK v1.90b3.45 (8), as described below:

1. Variants with MAC = 0, nr of alternative alleles ≠ 2 and call rate < 95% were filtered out (in vcftools);
2. Samples were filtered out when they showed:
   1. IBD sharing fraction (AJK) > 0.65 and coefficient of relantionship (ϕ) > 0.35 (computed through –relatedness and –relatedness2 in vcftools);
   2. relationships discrepant with the pedigree-based info - namely AJK ≥ 0.1, ϕ ≥ 0.083 and not showing the same family IDs, or AJK < 0.1, ϕ > 0.083 and showing the same family, maternal and paternal IDs;
   3. sample call rate < 90%;
   4. mismatch between sex reported in the pedigree and sex inferred from genetic data (through PLINK --check-sex command, default settings);
   5. genome-wide homozygosity outliers (|F| > 3 SDs from the mean of the distribution in the callset). F was computed through –het command in vcftools;
   6. samples showing an excess/depletion of singletons (absolute value of singletons count > 3 SDs from the mean of the distribution in the callset). Singletons were detected through –singletons command in vcftools.

123 PD cases (52 FPD + 71 SPD) and 334,671 variants (321,967 SNPs + 12,704 indels) passed QC. A multidimensional scaling (MDS) analysis carried out in PLINK confirmed the ancestry of the different subsets in our callset. After QC, mean (SD) depth per sample was 131 (26), while mean depth per locus was 131 (87). Variants passing QC were annotated to genes within 10 kb from transcription start/stop site, through Annovar version 1-2-2016 (9) and Ensembl Variant Effect Predictor (VEP) v88 (10). Variant annotation contained information concerning variant type, alternative allele frequency in the general population, and predicted effect on gene function.

*Molecular validation and genotyping of prioritized variants*

Prioritized variants were validated through Sanger sequencing or, alternatively, Polymerase Chain Reaction (PCR). For the case-control analysis, we genotyped the most represented variants in our cohort of patients for each of the VEP functional annotation class selected, namely rs772162369 in *MFSD6L* and rs56407180 in *KALRN* among HIGH variants (AF=2.03%), and rs201330591 in *GTF2H2* among MODERATE variants (AF=4.66%). Genotyping was performed using custom TaqMan® assays (Bio-Rad, USA) and analysed in a Bio-Rad® CFX96TM Real Time PCR detection system. We performed a general quality control of genotyped samples and variants, in PLINK. Samples showed good call rates, with only seven (out of 657) samples showing one missing call, out of three variants analysed. Similarly, variants were checked for call rate and Hardy Weinberg Equilibrium, both in all and in unaffected subjects only. None of these variants showed a call rate < 99.5%. This suggested the good quality of genotyping and justified the genetic association analysis of all the genotyped variants.

*Replication attempt in the IPDGC cohort*

We attempted a replication of the significant association observed in our dataset, in an independent case-control WES study of 1,148 young-onset unrelated PD cases (average age at onset 40.6 years; range 35–56 years) and 503 control participants of European ancestry, from the International Parkinson’s Disease Genomics Consortium (IPDGC) (11). We computed reference and alternative allele counts of rs201330591 and performed an allelic Fisher Exact Test on these counts, as above. Then we meta-analysed the resulting association with that observed in the Italian cohort, through a Mantel-Haenszel meta-analysis (meta.MH function of rmeta package in R; see URLs). This fixed-effect method shows optimal properties for comparing the OR of rare events (12), such as alternative allele counts in WES analyses.

*Genetic association test with continuous scales assessing PD symptoms*

UPDRS, MoCA and NMS scales elaborated as above were used for genetic association analysis of common variants. To this purpose, we removed subjects recruited in the ICP-Milan center (for whom no continuous measure was available), and for each of the scores analysed we set to missing phenotypic outliers, defined as samples showing a Z-score > 3 SDs below or above the mean of each measure. We carried out an additional quality control to filter out variants with Minor Allele Frequency < 5%, Hardy Weinberg Equilibrium p < 10^-6^, and those located in non-autosomal chromosomes. No sample showed an individual call rate below 5%. After QC, we tested for association 110,803 autosomal variants in 113 PD cases, with UPDRS (N=100), MoCA (N=96) and NMS scales (N=98). Association testing was performed in two steps. First, we performed univariate linear mixed effect models in EMMAX (version March 2010) (13), using cross-samples kinship (Balding-Nichols) matrix as random effect and different covariates as fixed effects, which included PD familiarity, sex, age, pharmacological treatment state at the time of phenotypic assessment (ON/OFF), duration of disease (years since diagnosis), daily L-Dopa dosage (mg/day) and the first 10 genetic ancestry (MDS) components, computed in the dataset which was involved in this analysis. Then, we carried out a multivariate genetic association study on all the three scales together, through TATES (14). This software combines the p-values obtained in univariate genetic association analysis on multiple (correlated) phenotypes, to produce one multivariate association p-value per SNP, while correcting for their cross-trait correlations (Table S5b). This method is optimal for detecting multivariate genetic associations affecting some, but not necessarily all, of a set of correlated phenotypes, and is also powerful in the detection of contrasting genetics effects (e.g. to identify SNPs affecting some phenotypes positively, some negatively) (14).

To follow-up on the results of the exome-wide association study, we genotyped the top hit identified in the exome-wide analysis (rs3835072) through a custom TaqMan® assay, as above, and carried out a quality control of the genotype calls generated, through PLINK. This variant showed a call rate > 99%, a Minor Allele Frequency of 37%, and was in Hardy-Weinberg Equilibrium (p = 0.46). Then we performed association analyses with continuous PD-related scales in the full Neuromed cohort (N=472), through linear association models with adaptive permutations in PLINK, using the same covariates as above except for genetic ancestry (since no genome-wide genetic data were available in the whole cohort). As above, we then combined the results of the univariate association tests into a multivariate association analysis through TATES.

**a)**

| **Recruiting**  **Center** | **Set** | **N**  **(families)** | **Age**  **(mean ± SD)** | **AAO**  **(mean ± SD)** | **Disease duration**  **(mean ± SD)** | **Sex ratio**  **(M/F/missing)** | **Familiarity**  **(FPD/SPD/missing)** | **PD Phenotype**  **(rigid-bradykinetic/tremorigenic/mixed/missing)** |
| --- | --- | --- | --- | --- | --- | --- | --- | --- |
| IRCCS  Neuromed | Total | 472 (458) | 66.63 ± 8.82 | 58.28 ± 9.98 | 8.27 ± 6.28 | 288/184/0 | 196/273/3 | 304/72/77/19 |
|  | FPD | 196 (183) | 66.20 ± 8.97 | 57.60 ± 10.50 | 8.58 ± 6.87 | 118/67/0 | - | 127/34/35/10 |
|  | SPD | 273 (273) | 67.00 ± 8.66 | 58.82 ± 9.59 | 8.05 ± 5.83 | 161/112/0 | - | 176/48/41/8 |
| ICP Milan | Total  (FPD) | 82 (42) | 66.7 ± 10.39 | 60.69 ± 10.62 | 6.32 ± 4.59 | 41/40/1 | 82/0/0 | Not available |

**b)**

| **Recruiting**  **Center** | **Set** | **N**  **(families)** | **Age**  **(mean ± SD)** | **AAO**  **(mean ± SD)** | **Disease duration**  **(mean ± SD)** | **Sex ratio**  **(M/F/missing)** | **Familiarity**  **(FPD/SPD/missing)** | **PD Phenotype**  **(rigid-bradykinetic/tremorigenic/mixed/missing)** |
| --- | --- | --- | --- | --- | --- | --- | --- | --- |
| IRCCS Neuromed | Total | 114 (110) | 65.08 ± 8.83 | 55.89 ± 9.98 | 9.22 ± 5.41 | 70/44/0 | 72/42/0 | 57/24/26/6 |
|  | FPD | 42 (38) | 63.31 ± 8.39 | 53.68 ± 10.57 | 9.75 ± 6.54 | 25/17/0 | - | 21/7/11/3 |
|  | SPD | 72 (72) | 66.13 ± 8.98 | 57.16 ± 9.46 | 8.91 ± 4.67 | 45/27/0 | - | 36/17/15/3 |
| ICP Milan | Total  (FPD) | 46 (23) | 68.31 ± 8.56 | 61.71 ± 8.57 | 6.46 ± 4.90 | 21/24/1 | 46/0/0 | Not available |

**Table S1.** Descriptive of **a)** the PD cohorts recruited and of **b)** the participants actually involved in the WES analysis presented in the paper. Age, Age at Onset and disease duration are reported in years. Abbreviations: FPD/SPD = Familal/Sporadic Parkinson Disease; AAO = Age at onset.

| **Severity Rank** | **Name** | **Description** | **IMPACT** |
| --- | --- | --- | --- |
| 1 | Transcript ablation | A feature ablation whereby the deleted region includes a transcript feature | HIGH |
| 2 | Splice acceptor variant | A splice variant that changes the 2 base region at the 3' end of an intron | HIGH |
| 3 | Splice donor variant | A splice variant that changes the 2 base region at the 5' end of an intron | HIGH |
| 4 | Stop gained | A sequence variant whereby at least one base of a codon is changed, resulting in a premature stop codon, leading to a shortened transcript | HIGH |
| 5 | Frameshift variant | A sequence variant which causes a disruption of the translational reading frame, because the number of nucleotides inserted or deleted is not a multiple of three | HIGH |
| 6 | Stop lost | A sequence variant where at least one base of the terminator codon (stop) is changed, resulting in an elongated transcript | HIGH |
| 7 | Start lost | A codon variant that changes at least one base of the canonical start codon | HIGH |
| 8 | Transcript amplification | A feature amplification of a region containing a transcript | HIGH |
| 9 | Inframe insertion | An inframe non synonymous variant that inserts bases into in the coding sequence | MODERATE |
| 10 | Inframe deletion | An inframe non synonymous variant that deletes bases from the coding sequence | MODERATE |
| 11 | Missense variant | A sequence variant, that changes one or more bases, resulting in a different amino acid sequence but where the length is preserved | MODERATE |
| 12 | Protein altering variant | A sequence variant which is predicted to change the protein encoded in the coding sequence | MODERATE |

**Table S2.** Classification of variants of HIGH and MODERATE impact on protein function, by the Ensembl Variant Effect Predictor (VEP) v88 (10).

**a)**

| Variant  (Chr:bp) | REF  allele | ALT  allele | AF  (%) | AC | AN | HOM.REF | HET | HOM.ALT | Location  (consequence) | Gene | 1000G EUR  (%) | ESP EA  (%) | ExAC NFE  (%) |
| --- | --- | --- | --- | --- | --- | --- | --- | --- | --- | --- | --- | --- | --- |
| **rs772162369 (17:8701167)** | **CA** | **C** | **2.03** | **5** | **246** | **119** | **3** | **1** | **exonic**  **(frameshift deletion)** | **MFSD6L** | **0.00** | **0.22** | **0.15** |
| **rs56407180 (3:124303696)** | **C** | **T** | **2.03** | **5** | **246** | **118** | **5** | **0** | **exonic**  **(stop gain)** | **KALRN** | **0.10** | **0.15** | **0.39** |
| rs766441817 (19:43420567) | GA | G | 1.63 | 4 | 246 | 119 | 4 | 0 | exonic  (frameshift) | PSG6 | 0.00 | 0.00 | 0.2 |
| rs759514260 (16:1779569) | CCGGGGCGGAGG  TACGCGGGGCGCG  GCGGGGTGGAGG  TACGCGGGGCGCGG | C | 1.63 | 4 | 246 | 119 | 4 | 0 | exonic  (splicing) | MAPK8IP3 | 0.00 | 0.00 | 0.2 |
| rs147792046 (2:175263171) | G | T | 1.63 | 4 | 246 | 119 | 4 | 0 | intronic  (splicing) | SCRN3 | 0.00 | 0.07 | 0.08 |
| rs146935160 (4:77230620) | A | T | 1.22 | 3 | 246 | 120 | 3 | 0 | exonic  (stop gain) | FAM47E-STBD1,  STBD1 | 0.10 | 0.00 | 0.15 |
| rs376367532 (15:101601485) | GCCACCGAGGTAAGCAC  TGCCCGCAGGCCTGC | G | 1.22 | 3 | 246 | 120 | 3 | 0 | exonic  (splicing) | LRRK1 | 0.10 | 0.00 | 0.09 |

**b)**

| Variant (chr:bp) | REF | ALT | AF  (%) | AC | AN | HOM.REF | HET | HOM.ALT | Location  (consequence) | Gene | 1000G EUR  (%) | ESP EA  (%) | ExAC NFE  (%) |
| --- | --- | --- | --- | --- | --- | --- | --- | --- | --- | --- | --- | --- | --- |
| **rs201330591**  **(5:70351261)** | **A** | **T** | **4.66** | **11** | **236** | **109** | **7** | **2** | **exonic**  **(missense)** | **GTF2H2** | **0.20** | **0.32** | **0.50** |
| rs143022124  (11:96117520) | C | T | 4.07 | 10 | 246 | 113 | 10 | 0 | exonic  (missense) | CCDC82 | 0.10 | 0.30 | 0.34 |
| rs535061628  (14:39901257) | CGTCGCAGCT | C | 4.07 | 10 | 246 | 113 | 10 | 0 | exonic  (missense) | FBXO33 | 0.20 | 0.00 | 0.00 |
| rs34080825  (5:38933482) | C | G | 4.07 | 10 | 246 | 113 | 10 | 0 | exonic  (missense) | OSMR | 0.60 | 0.72 | 0.81 |
| rs146569565  (19:43433676) | G | C | 4.07 | 10 | 246 | 113 | 10 | 0 | exonic  (missense) | PSG7 | 0.80 | 0.74 | 0.78 |
| rs79737301  (10:73121779) | T | C | 4.07 | 10 | 246 | 113 | 10 | 0 | exonic  (missense) | SLC29A3 | 0.70 | 0.64 | 0.72 |
| rs180870155  (19:4499597) | A | G | 4.07 | 10 | 246 | 113 | 10 | 0 | exonic  (missense) | HDGFRP2 | 0.80 | 0.52 | 0.60 |
| rs146685560  (6:30997275) | C | G | 4.07 | 10 | 246 | 113 | 10 | 0 | exonic  (missense) | MUC22 | 0.70 | 0.00 | 0.55 |
| rs45609739  (11:114400991) | G | C | 3.66 | 9 | 246 | 114 | 9 | 0 | exonic  (missense) | NXPE1 | 0.50 | 0.51 | 0.60 |
| rs75209396  (10:124330421) | C | G | 3.66 | 9 | 246 | 114 | 9 | 0 | exonic  (missense) | DMBT1 | 0.70 | 0.62 | 0.55 |
| rs112908035  (3:48419957) | A | G | 3.66 | 9 | 246 | 114 | 9 | 0 | exonic  (missense) | FBXW12 | 0.50 | 0.57 | 0.65 |
| rs79879036  (2:141092084) | T | G | 3.25 | 8 | 246 | 116 | 6 | 1 | exonic  (missense) | LRP1B | 0.50 | 0.59 | 0.48 |
| rs2231370  (6:3152760) | T | C | 3.25 | 8 | 246 | 116 | 6 | 1 | exonic  (missense) | BPHL | 0.50 | 0.36 | 0.35 |
| rs61744879  (5:79054599) | C | T | 3.25 | 8 | 246 | 115 | 8 | 0 | exonic  (missense) | CMYA5 | 0.10 | 0.26 | 0.36 |
| rs12407578  (1:12337667) | C | T | 3.25 | 8 | 246 | 115 | 8 | 0 | exonic  (missense) | VPS13D | 0.50 | 0.56 | 0.43 |
| rs144315661  (7:91503914) | G | A | 3.25 | 8 | 246 | 115 | 8 | 0 | exonic  (missense) | MTERF | 0.50 | 0.55 | 0.54 |
| rs150030843  (16:30004819) | C | T | 3.25 | 8 | 246 | 115 | 8 | 0 | exonic  (missense) | HIRIP3 | 0.20 | 0.29 | 0.39 |
| rs74514616  (14:106845487) | C | G | 3.25 | 8 | 246 | 115 | 8 | 0 | exonic  (missense) | IGHV3-35 | 0.20 | 0.24 | 0.30 |
| rs140756827  (14:68250147) | C | T | 2.85 | 7 | 246 | 116 | 7 | 0 | exonic  (missense) | ZFYVE26 | 0.40 | 0.37 | 0.40 |
| rs139092247  (15:78783019) | G | A | 2.85 | 7 | 246 | 117 | 5 | 1 | exonic  (missense) | IREB2 | 0.20 | 0.34 | 0.34 |

**Table S3.** Prioritized **a)** HIGH and **b)** MODERATE variants validated in our PD callset (N=123). Variants which were selected for targeted genotyping and case-control association testing in the whole Neuromed cohort are highlighted in bold.

Abbreviations: REF/ALT = reference/alternative allele; AF/AC = alternative allele frequency/count; AN = total allele number; HOM REF/ HET/ HOM ALT = reference homozygous / heterozygous / alternative homozygous genotype count; 1000G EUR = European Sample of the 1000 Genomes project, phase 3 (N=503) (15); ESP EA = NHLBI Exome Sequencing Project 6500 release si-v2, European ancestry (N=4,300) (16); ExAC NFE = Exome Aggregation Consortium version 0.3.1, Non-Finnish Europeans (N= 33,370) (17).

| **Chr** | **Bp** | **Variant**  **(dbSNP147)** | **REF** | **ALT** | **AF**  **(%)** | **AC** | **AN** | **HOM**  **REF** | **HET** | **HOM**  **ALT** | **Location**  **(Consequence)** | **Gene** | **1000G**  **EUR**  **(%)** | **ESP**  **EA**  **(%)** | **ExAC**  **NFE**  **(%)** | **VEP Impact** |
| --- | --- | --- | --- | --- | --- | --- | --- | --- | --- | --- | --- | --- | --- | --- | --- | --- |
| 17 | 8701167 | rs772162369 | CA | C | 2.03 | 5 | 246 | 119 | 3 | 1 | Exonic  (frameshift deletion) | MFSD6L | 0.00 | 0.22 | 0.15 | H |
| 3 | 124303696 | rs56407180 | C | T | 2.03 | 5 | 246 | 118 | 5 | 0 | Exonic  (stop gain) | KALRN | 0.10 | 0.15 | 0.39 | H |
| 5 | 70351261 | rs201330591 | A | T | 4.66 | 11 | 236 | 109 | 7 | 2 | Exonic  (missense) | GTF2H2 | 0.20 | 0.32 | 0.50 | M |

**Table S4.** Most frequent genetic variants detected through the bioinformatics pipeline in each of the two VEP functional annotation classes, namely HIGH and MODERATE impact variants (see Figure 1 and *Variants prioritization, validation and genetic association analysis with PD status* in the *Methods* section).

Abbreviations: REF/ALT = reference/alternative allele; AF/AC = alternative allele frequency/count; AN = total allele number; HOM REF/ HET/ HOM ALT = reference homozygous / heterozygous / alternative homozygous genotype count; 1000G EUR = European Sample of the 1000 Genomes project, phase 3 (N=503) (15); ESP EA = NHLBI Exome Sequencing Project 6500 release si-v2, European ancestry (N=4,300) (16); ExAC NFE = Exome Aggregation Consortium version 0.3.1, Non-Finnish Europeans (N= 33,370) (17); M/H = moderate/high impact on protein function (as predicted by VEP v88 (10)).

**a)**

| Descriptive | UPDRS | MoCA | NMS |
| --- | --- | --- | --- |
| N | 441 | 452 | 456 |
| missing | 31 | 20 | 16 |
| min | 0 | 0 | 0 |
| max | 72 | 30 | 203 |
| mean | 22.46 | 22.65 | 56.83 |
| median | 21 | 24 | 52.5 |
| SD | 11.47 | 5.96 | 35.34 |
| SE | 0.55 | 0.28 | 1.66 |

**b)**

| PD scale | UPDRS | MoCA | NMS |
| --- | --- | --- | --- |
| UPDRS | 1 | -0.41 | 0.34 |
| MoCA | [-0.49; -0.33] | 1 | -0.40 |
| NMS | [0.26; 0.43] | [-0.47; -0.31] | 1 |

**Table S5. a)** General descriptive statistics and **b)** cross-trait correlations of scales assessing motor and non-motor symptoms of PD in the Neuromed cohort (see *Phenotypic Assessment of PD cases* above for details). In **b)**, point Pearson´s r coefficients and the relevant 95% Confidence Intervals are reported in the upper and lower triangle of the matrix, respectively.

Abbreviations: UPDRS = Movement Disorder Society revised version of the Unified Parkinson’s Disease Rating Scale - Part III (2); MoCA = Montreal Cognitive Assessment (3); NMS = modified version of the Non Motor Symptoms Scale for Parkinson Disease (see above) (4); SD = standard deviation; SE = standard error.

**Supplementary Results**

| **Study** | **N (cases/controls)** | **OR** | **Low** | **Up** | **Variant (Gene)** | **Heterogeneity** |
| --- | --- | --- | --- | --- | --- | --- |
| Neuromed | 655  (445/210) | 8.16 | 1.08 | 61.52 | rs201330591  (GTF2H2) | χ^2^_1_ = 3.36 (0.067) |
| IPDGC | 714  (545/169) | 1.12 | 0.41 | 3.04 |  |  |
| Meta-analysis | 1369  (990/379) | 2.18 | 0.92 | 5.15 |  |  |

**Table S6.** Association with PD status of rs201330591 (*GTF2H2*; effect allele T). Here, we report association statistics (OR and 95% Confidence Interval) observed in the Neuromed and in the IPGDC study (11), along with Mantel-Haenszel meta-analysis results.

**a)**

**
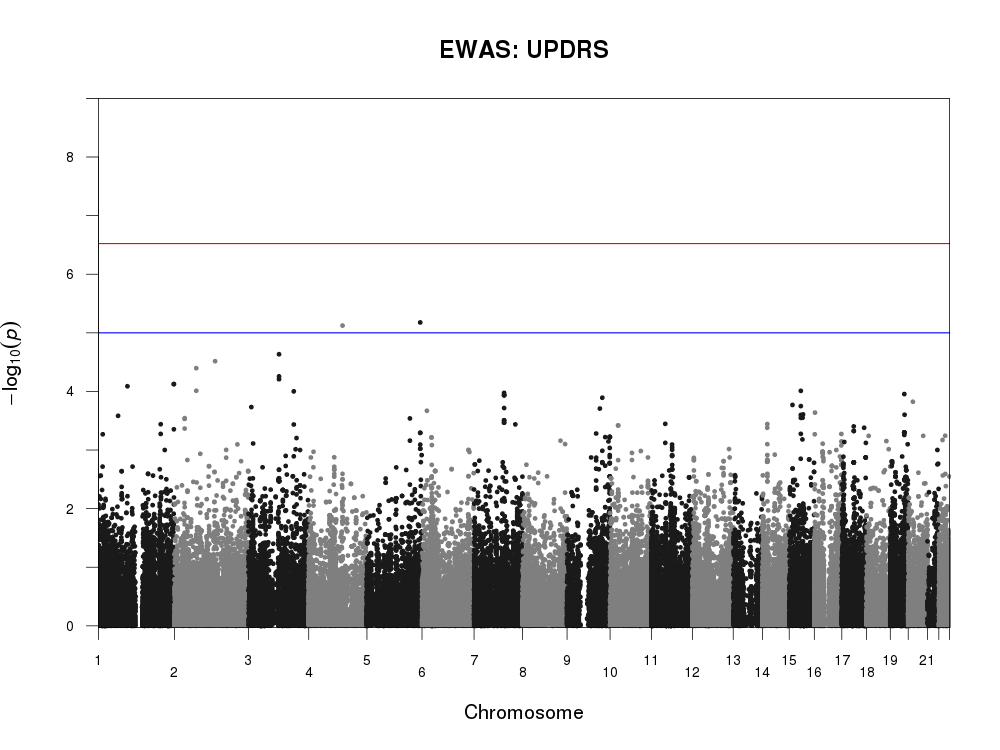
**

**b)**

**
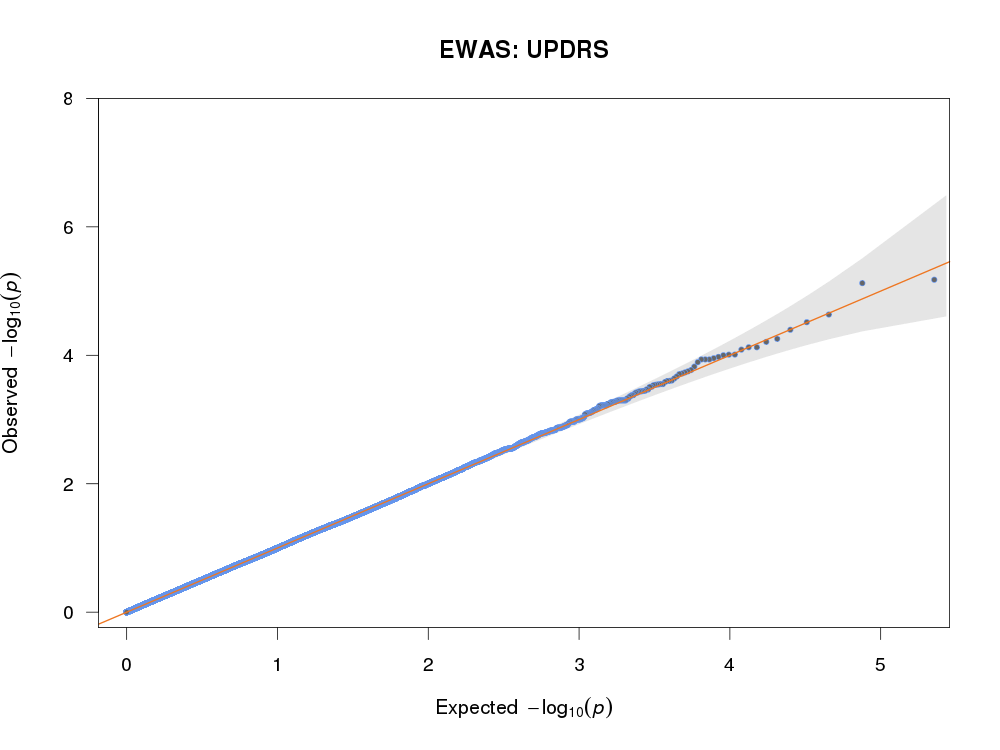
**

**c)**

**
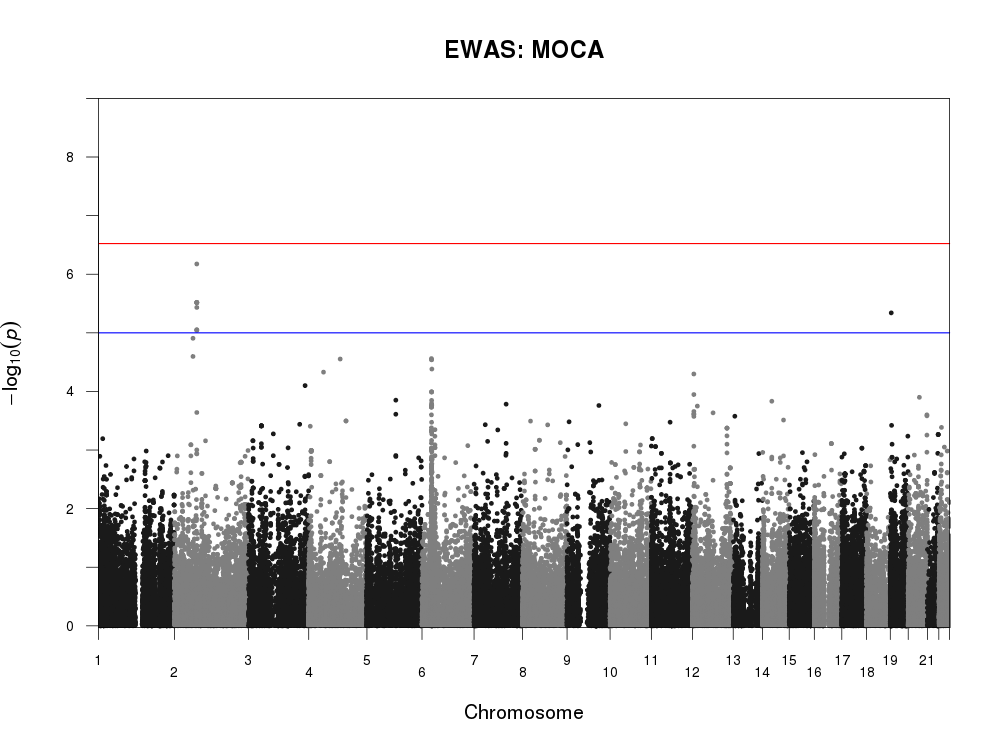
**

**d)**

**
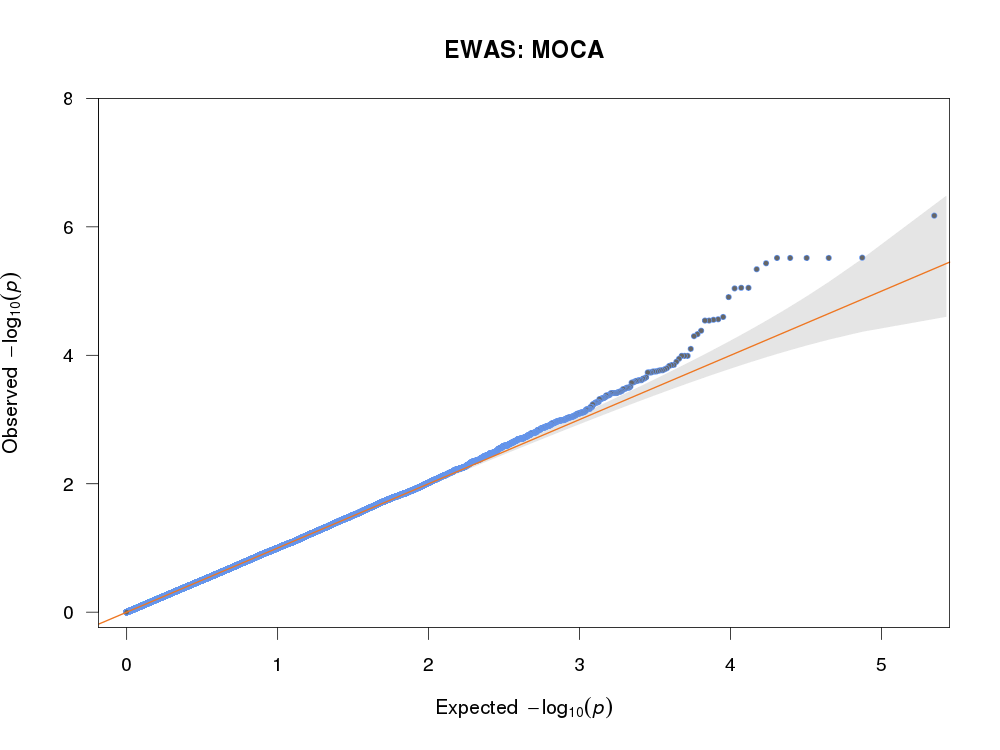
**

**e)**

**
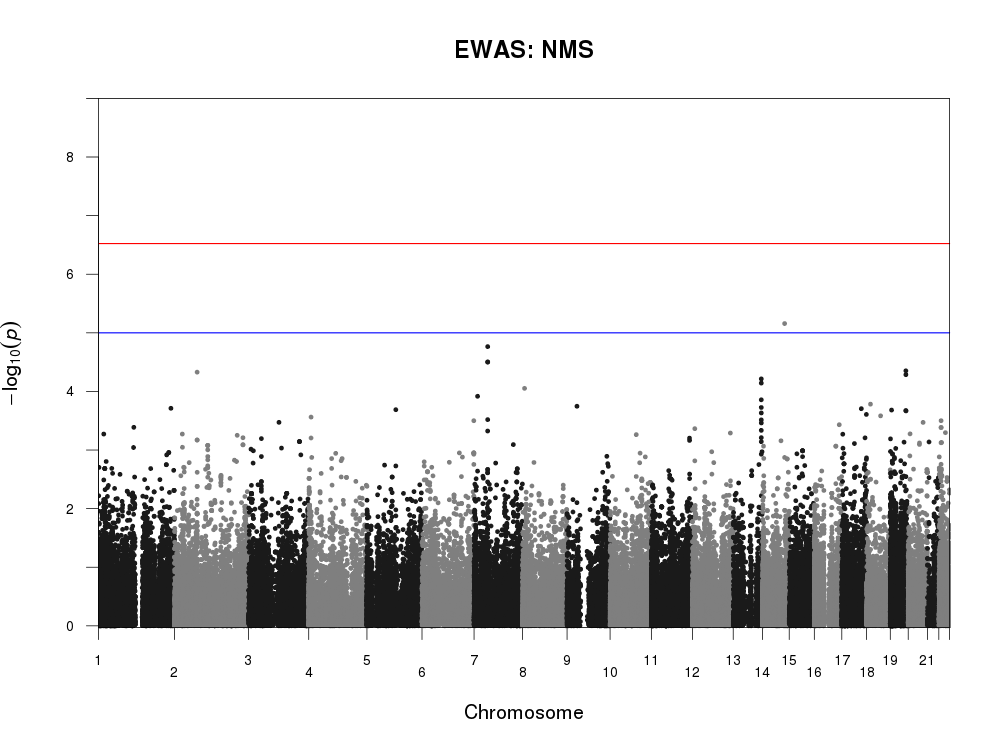
**

**f)**

**
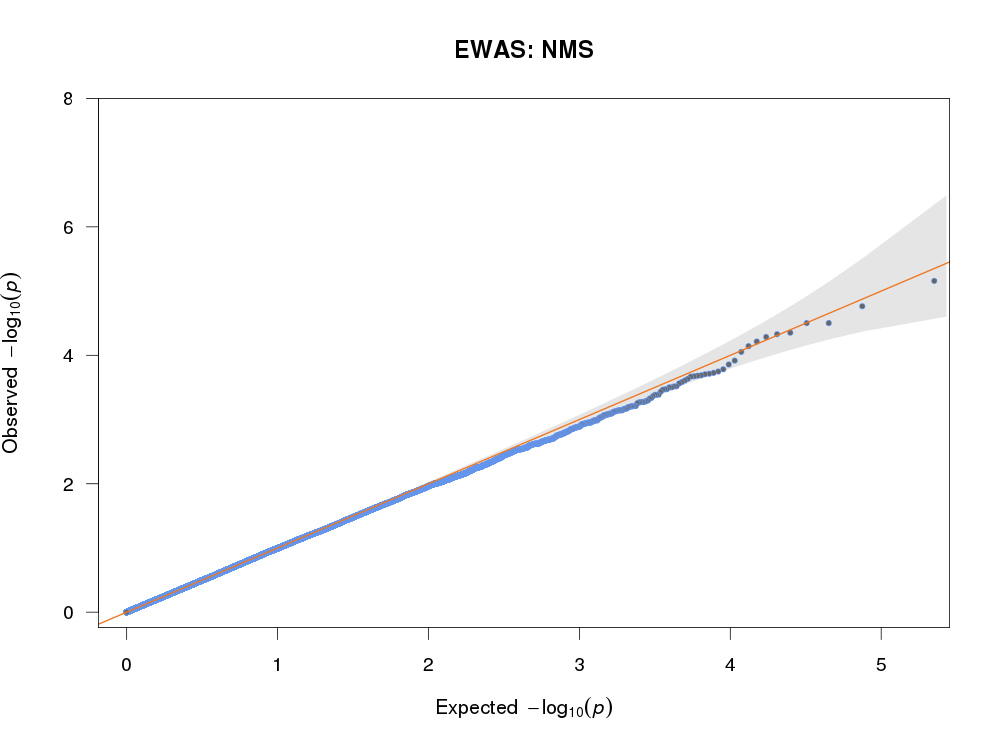
**

**g)**

**
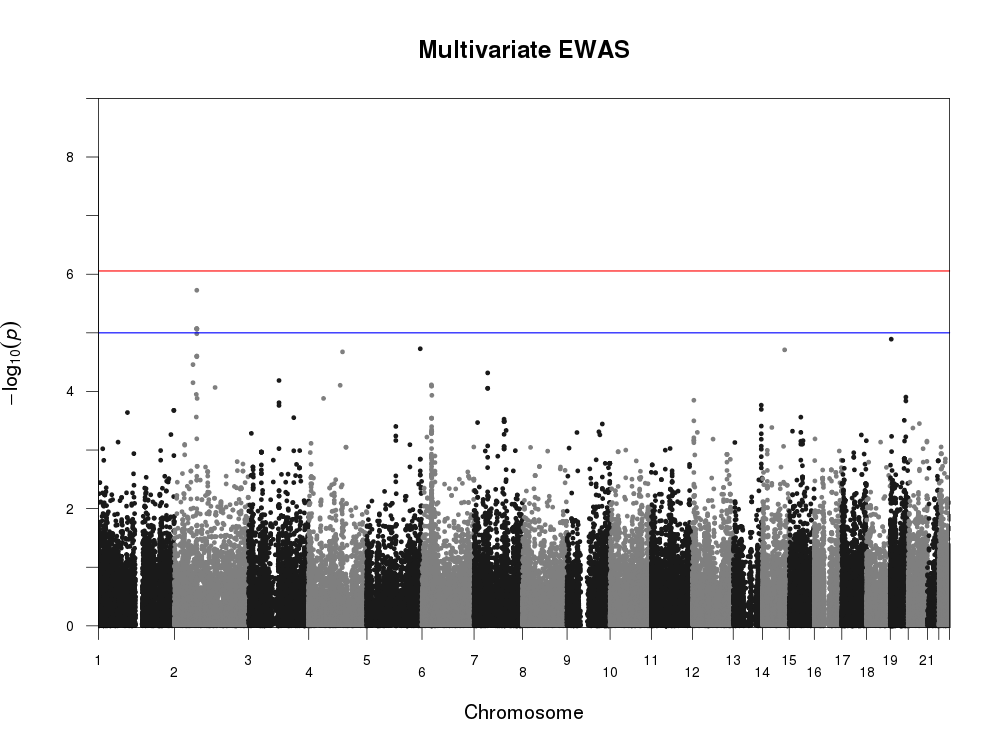
**

**h)**

**
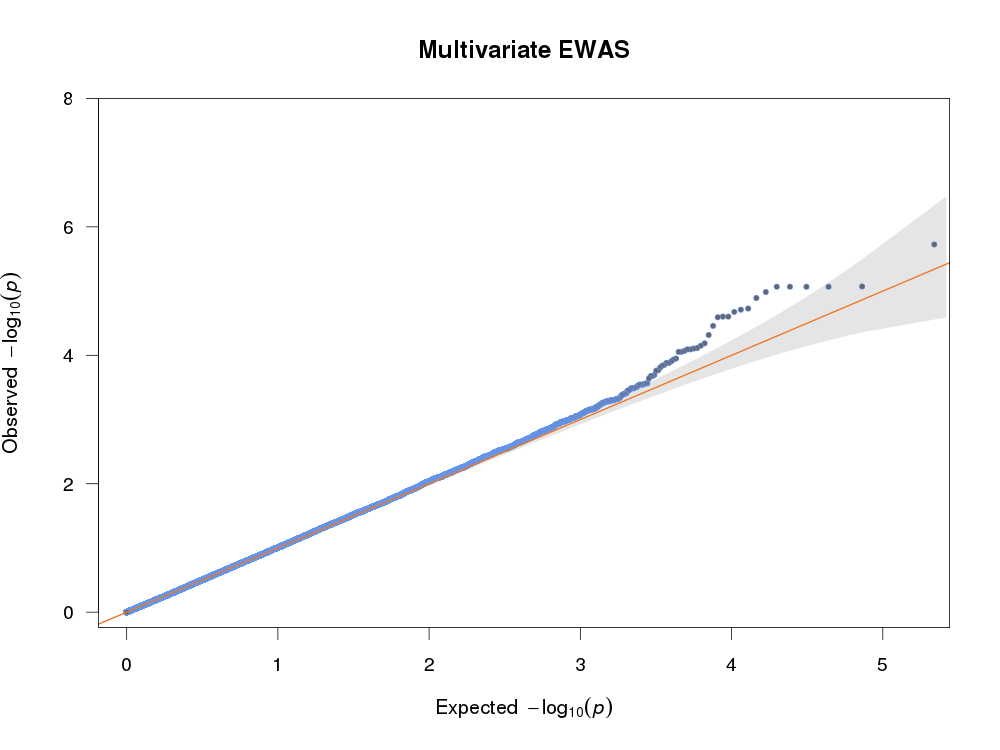
**

**Figure S1.** Manhattan and QQ plots of the Exome-Wide Association Scans (EWAS) carried out in the present study. **a-b)** UPDRS (Movement Disorder Society revised version of the Unified Parkinson’s Disease Rating Scale - Part III) (2); **c-d)** MoCA (Montreal Cognitive Assessment (3); **e-f)** NMS (modified version of Non Motor Symptoms Scale for Parkinson Disease; see *Phenotypic Assessment of PD cases* above for details) (4); **g-h)** multivariate association analysis of the three scales. Blue and red lines indicate suggestive (α = 10^-5^) and exome-wide significance thresholds (α = 2.95x10^-7^ and 8.84x10^-7^, for univariate and multivariate EWAS, respectively). The latter is corrected for the number of LD-independent SNPs tested in the multivariate EWAS (56,588, as computed by the Genetic Type I error calculator) (18), and also for the number of independent scales tested (3) in the univariate EWAS analyses.

| SNP | UPDRS^a^ | MoCA^a^ | NMS^a^ | Multivariate^b^ |
| --- | --- | --- | --- | --- |
| rs3835072 | 0.03  (-0.038) | 6.7 x 10^-7^  (0.090) | 0.42  (-0.011) | 1.9 x 10^-6^ |
| rs1864492 | 0.37  (-0.018) | 3.0 x 10^-6^ (0.094) | 0.73  (-0.005) | 8.5 x 10^-6^ |
| rs2288631 | 0.09  (-0.030) | 3.1 x 10^-6^ (0.084) | 0.54  (-0.008) | 8.6 x 10^-6^ |
| rs1053329 | 0.09  (-0.030) | 3.1 x 10^-6^ (0.084) | 0.54  (-0.008) | 8.6 x 10^-6^ |
| rs7851 | 0.09  (-0.030) | 3.1 x 10^-6^ (0.084) | 0.54  (-0.008) | 8.6 x 10^-6^ |
| rs909065 | 0.09  (-0.030) | 3.1 x 10^-6^ (0.084) | 0.54  (-0.008) | 8.6 x 10^-6^ |

**Table S7.** Most significant genetic associations (p < 10^-5^) detected in the multivariate Exome Wide Association Scan of three continuous scales assessing PD-related symptoms (see abbreviations below). No variants met exome-wide significance thresholds, neither in the univariate (α = 2.95x10^-7^), nor in the multivariate EWAS (α = 8.84x10^-7^).

^a^ Univariate association p-values as computed by EMMAX linear mixed model are reported, along with betas (β) values referring to major allele (A1) in brackets.

^b^ Multivariate association p-values as computed by the software TATES are reported (no β value was produced in the output).

| Scale | P | Beta | SE | N | Multivariate P |
| --- | --- | --- | --- | --- | --- |
| UPDRS | 0.17 | -0.013 | 0.010 | 394 | 0.19 |
| MOCA | 0.07 | 0.018 | 0.010 | 395 |  |
| NMS | 0.67 | -0.003 | 0.006 | 410 |  |

**Table S8.** Univariate and multivariate association statistics of rs3835072 with the three PD-related scales analysed in the paper. Univariate p-values, betas (β) and relevant Standard Errors (SE) are reported, along with the actual sample size of the analysis for each scale. For multivariate analysis, only the p-value is reported, as per TATES output (14).

Abbreviations: UPDRS = Movement Disorder Society revised version of the Unified Parkinson’s Disease Rating Scale - Part III (2); MoCA = Montreal Cognitive Assessment (3); NMS = modified version of the Non Motor Symptoms Scale for Parkinson Disease (see above) (4).

**References**

1. Postuma RB, Berg D, Stern M, Poewe W, Olanow CW, Oertel W, Obeso J, Marek K, Litvan I, Lang AE, et al. MDS clinical diagnostic criteria for Parkinson’s disease. *Mov Disord* (2015) **30**:1591–1601. doi:10.1002/mds.26424

2. Hentz JG, Mehta SH, Shill HA, Driver-Dunckley E, Beach TG, Adler CH. Simplified conversion method for unified Parkinson’s disease rating scale motor examinations. *Mov Disord* (2015) **30**:1967–1970. doi:10.1002/mds.26435

3. Conti S, Bonazzi S, Laiacona M, Masina M, Coralli MV. Montreal Cognitive Assessment (MoCA)-Italian version: regression based norms and equivalent scores. *Neurol Sci* (2015) **36**:209–214. doi:10.1007/s10072-014-1921-3

4. Cova I, Di Battista ME, Vanacore N, Papi CP, Alampi G, Rubino A, Valente M, Meco G, Contri P, Di Pucchio A, et al. Validation of the Italian version of the Non Motor Symptoms Scale for Parkinson’s disease. *Park Relat Disord* (2017) **34**:38–42. doi:10.1016/j.parkreldis.2016.10.020

5. Li H, Durbin R. Fast and accurate short read alignment with Burrows-Wheeler transform. *Bioinformatics* (2009) **25**:1754–1760. doi:10.1093/bioinformatics/btp324

6. DePristo MA, Banks E, Poplin R, Garimella K V, Maguire JR, Hartl C, Philippakis AA, del Angel G, Rivas MA, Hanna M, et al. A framework for variation discovery and genotyping using next-generation DNA sequencing data. *Nat Genet* (2011) **43**:491. Available at: https://doi.org/10.1038/ng.806

7. Danecek P, Auton A, Abecasis G, Albers C, Banks E, DePristo M. The variant call format and vcftools. *Bioinformatics* (2011) **27**: doi:10.1093/bioinformatics/btr330

8. Chang CC, Chow CC, Tellier LCAM, Vattikuti S, Purcell SM, Lee JJ. Second-generation PLINK: rising to the challenge of larger and richer datasets. *Gigascience* (2015) **4**:7. doi:10.1186/s13742-015-0047-8

9. Wang K, Li M, Hakonarson H. ANNOVAR: functional annotation of genetic variants from high-throughput sequencing data. *Nucleic Acids Res* (2010) **38**:e164. doi:10.1093/nar/gkq603

10. McLaren W, Gil L, Hunt SE, Riat HS, Ritchie GRS, Thormann A, Flicek P, Cunningham F. The Ensembl Variant Effect Predictor. *Genome Biol* (2016) **17**:122. doi:10.1186/s13059-016-0974-4

11. Jansen IE, Ye H, Heetveld S, Lechler MC, Michels H, Seinstra RI, Lubbe SJ, Drouet V, Lesage S, Majounie E, et al. Discovery and functional prioritization of Parkinson’s disease candidate genes from large-scale whole exome sequencing. *Genome Biol* (2017) **18**:1–26. doi:10.1186/s13059-017-1147-9

12. Collaboration. TC. *Cochrane Handbook for Systematic Reviews of Interventions Version 5.1.0*. updated Ma. , eds. J. Higgins, S. Green (2011). Available at: www.handbook.cochrane.org

13. Kang HM, Sul JH, Service SK, Zaitlen NA, Kong S-Y, Freimer NB, Sabatti C, Eskin E. Variance component model to account for sample structure in genome-wide association studies. *Nat Genet* (2010) **42**:348–354. doi:10.1038/ng.548

14. van der Sluis S, Posthuma D, Dolan C V. TATES: Efficient Multivariate Genotype-Phenotype Analysis for Genome-Wide Association Studies. *PLOS Genet* (2013) **9**:e1003235. Available at: https://doi.org/10.1371/journal.pgen.1003235

15. The Genomes Project C. A global reference for human genetic variation. *Nature* (2015) **526**:68. doi:10.1038/nature15393https://www.nature.com/articles/nature15393#supplementary-information

16. Auer PL, Reiner AP, Wang G, Kang HM, Abecasis GR, Altshuler D, Bamshad MJ, Nickerson DA, Tracy RP, Rich SS, et al. Guidelines for Large-Scale Sequence-Based Complex Trait Association Studies: Lessons Learned from the NHLBI Exome Sequencing Project. *Am J Hum Genet* (2016) **99**:791–801. doi:10.1016/j.ajhg.2016.08.012

17. Lek M, Karczewski KJ, Minikel E V, Samocha KE, Banks E, Fennell T, O’Donnell-Luria AH, Ware JS, Hill AJ, Cummings BB, et al. Analysis of protein-coding genetic variation in 60,706 humans. *Nature* (2016) **536**:285. Available at: https://doi.org/10.1038/nature19057

18. Li M-X, Yeung JMY, Cherny SS, Sham PC. Evaluating the effective numbers of independent tests and significant p-value thresholds in commercial genotyping arrays and public imputation reference datasets. *Hum Genet* (2012) **131**:747–756. doi:10.1007/s00439-011-1118-2

**URLs**

Annovar: <http://annovar.openbioinformatics.org/en/latest/>

Variant Effect Predictor (VEP): <https://www.ensembl.org/info/docs/tools/vep/index.html>

Genome Analysis Toolkit (GATK): <https://software.broadinstitute.org/gatk/>

Burrows Wheeler Aligner (BWA): <http://bio-bwa.sourceforge.net/>

Samtools: <http://samtools.sourceforge.net/>

Picard: <http://broadinstitute.github.io/picard>

Vcftools: <https://vcftools.github.io/index.html>

PLINK: <https://www.cog-genomics.org/plink/1.9/>

1000 Genomes Project: <ftp://ftp.1000genomes.ebi.ac.uk/vol1/ftp/>

NHLBI Exome Sequencing Project: <http://eversusgs.washington.edu/EVS/>

Exome Aggregation Consortium: <http://exac.broadinstitute.org/>

Rmeta package: <https://cran.r-project.org/web/packages/rmeta/index.html>

EMMAX: <http://genetics.cs.ucla.edu/emmax/index.html>

TATES: <https://ctg.cncr.nl/software/tates>

GEC: <http://grass.cgs.hku.hk/gec/>

**IPDGC consortium members and affiliations:**

**United Kingdom:** Alastair J Noyce (Preventive Neurology Unit, Wolfson Institute of Preventive Medicine, QMUL, London, UK and Department of Molecular Neuroscience, UCL, London, UK), Rauan Kaiyrzhanov (Department of Molecular Neuroscience, UCL Institute of Neurology, London, UK), Ben Middlehurst (Institute of Translational Medicine, University of Liverpool, Liverpool, UK), Demis A Kia (UCL Genetics Institute; and Department of Molecular Neuroscience, UCL Institute of Neurology, London, UK), Manuela Tan (Department of Clinical Neuroscience, University College London, London, UK), Henry Houlden (Department of Molecular Neuroscience, UCL Institute of Neurology, London, UK),  Huw R Morris (Department of Clinical Neuroscience, University College London, London, UK), Helene Plun-Favreau (Department of Molecular Neuroscience, UCL Institute of Neurology, London, UK), Peter Holmans (Biostatistics & Bioinformatics Unit, Institute of Psychological Medicine and Clinical Neuroscience, MRC Centre for Neuropsychiatric Genetics & Genomics, Cardiff, UK), John Hardy (Department of Molecular Neuroscience, UCL Institute of Neurology, London, UK), Daniah Trabzuni (Department of Molecular Neuroscience, UCL Institute of Neurology, London, UK; Department of Genetics, King Faisal Specialist Hospital and Research Centre, Riyadh, 11211 Saudi Arabia), John Quinn (Institute of Translational Medicine, University of Liverpool, Liverpool, UK), Vivien Bubb (Institute of Translational Medicine, University of Liverpool, Liverpool, UK), Kin Y Mok (Department of Molecular Neuroscience, UCL Institute of Neurology, London, UK), Kerri J. Kinghorn (Institute of Healthy Ageing, University College London, London, UK), Kimberley Billingsley (Institute of Translational Medicine, University of Liverpool, Liverpool, UK), Nicholas W Wood (UCL Genetics Institute; and Department of Molecular Neuroscience, UCL Institute of Neurology, London, UK), Patrick Lewis (University of Reading, Reading, UK), Sebastian Schreglmann (Department of Molecular Neuroscience, UCL Institute of Neurology, London, UK), Ruth Lovering (University College London, London, UK), Lea R’Bibo (Department of Molecular Neuroscience, UCL Institute of Neurology, London, UK), Claudia Manzoni (University of Reading, Reading, UK), Mie Rizig (Department of Molecular Neuroscience, UCL Institute of Neurology, London, UK), Mina Ryten (Department of Molecular Neuroscience, UCL Institute of Neurology, London, UK), Sebastian Guelfi (Department of Molecular Neuroscience, UCL Institute of Neurology, London, UK), Valentina Escott-Price (MRC Centre for Neuropsychiatric Genetics and Genomics, Cardiff University School of Medicine, Cardiff, UK), Viorica Chelban (Department of Molecular Neuroscience, UCL Institute of Neurology, London, UK), Thomas Foltynie (UCL Institute of Neurology, London, UK), Nigel Williams (MRC Centre for Neuropsychiatric Genetics and Genomics, Cardiff, UK), Karen E. Morrison (Faculty of Medicine, University of Southampton, UK), Carl Clarke (University of Birmingham, Birmingham, UK and Sandwell and West Birmingham Hospitals NHS Trust, Birmingham, UK).

**France:** Alexis Brice (Institut du Cerveau et de la Moelle épinière, ICM, Inserm U 1127, CNRS, UMR 7225, Sorbonne Universités, UPMC University Paris 06, UMR S 1127, AP-HP, Pitié-Salpêtrière Hospital, Paris, France), Fabrice Danjou (Institut du Cerveau et de la Moelle épinière, ICM, Inserm U 1127, CNRS, UMR 7225, Sorbonne Universités, UPMC University Paris 06, UMR S 1127, AP-HP, Pitié-Salpêtrière Hospital, Paris, France), Suzanne Lesage (Institut du Cerveau et de la Moelle épinière, ICM, Inserm U 1127, CNRS, UMR 7225, Sorbonne Universités, UPMC University Paris 06, UMR S 1127, AP-HP, Pitié-Salpêtrière Hospital, Paris, France), Jean-Christophe Corvol (Institut du Cerveau et de la Moelle épinière, ICM, Inserm U 1127, CNRS, UMR 7225, Sorbonne Universités, UPMC University Paris 06, UMR S 1127, Centre d’Investigation Clinique Pitié Neurosciences CIC-1422, AP-HP, Pitié-Salpêtrière Hospital, Paris, France), Maria Martinez (INSERM UMR 1220; and Paul Sabatier University, Toulouse, France),

**Germany:** Claudia Schulte (Department for Neurodegenerative Diseases, Hertie Institute for Clinical Brain Research, University of Tübingen, and DZNE, German Center for Neurodegenerative Diseases, Tübingen, Germany), Kathrin Brockmann (Department for Neurodegenerative Diseases, Hertie Institute for Clinical Brain Research, University of Tübingen, and DZNE, German Center for Neurodegenerative Diseases, Tübingen, Germany), Javier Simón-Sánchez (Department for Neurodegenerative Diseases, Hertie Institute for Clinical Brain Research, University of Tübingen, and DZNE, German Center for Neurodegenerative Diseases, Tübingen, Germany), Peter Heutink (DZNE, German Center for Neurodegenerative Diseases and Department for Neurodegenerative Diseases, Hertie Institute for Clinical Brain Research, University of Tübingen, Tübingen, Germany), Patrizia Rizzu  (DZNE, German Center for Neurodegenerative Diseases), Manu Sharma (Centre for Genetic Epidemiology, Institute for Clinical Epidemiology and Applied Biometry, University of Tubingen, Germany), Thomas Gasser (Department for Neurodegenerative Diseases, Hertie Institute for Clinical Brain Research, and DZNE, German Center for Neurodegenerative Diseases, Tübingen, Germany),

**United States of America:** Mark R Cookson (Laboratory of Neurogenetics, National Institute on Aging, Bethesda, USA), Sara Bandres-Ciga (Laboratory of Neurogenetics, National Institute on Aging, Bethesda, MD, USA), Cornelis Blauwendraat (National Institute on Aging and National Institute of Neurological Disorders and Stroke, USA), David W. Craig (Department of Translational Genomics, Keck School of Medicine, University of Southern California, Los Angeles, USA), Derek Narendra (Inherited Movement Disorders Unit, National Institute of Neurological Disorders and Stroke, Bethesda, MD, USA), Faraz Faghri (Laboratory of Neurogenetics, National Institute on Aging, Bethesda, USA; Department of Computer Science, University of Illinois at Urbana-Champaign, Urbana, IL, USA), J Raphael Gibbs (Laboratory of Neurogenetics, National Institute on Aging, National Institutes of Health, Bethesda, MD, USA), Dena G. Hernandez (Laboratory of Neurogenetics, National Institute on Aging, Bethesda, MD, USA), Kendall Van Keuren-Jensen (Neurogenomics Division, TGen, Phoenix, AZ USA), Joshua M. Shulman (Departments of Neurology, Neuroscience, and Molecular & Human Genetics, Baylor College of Medicine, Houston, Texas, USA; Jan and Dan Duncan Neurological Research Institute, Texas Children’s Hospital, Houston, Texas, USA), Hirotaka Iwaki (Laboratory of Neurogenetics, National Institute on Aging, Bethesda, MD, USA), Hampton L. Leonard (Laboratory of Neurogenetics, National Institute on Aging, Bethesda, MD, USA),  Mike A. Nalls (Laboratory of Neurogenetics, National Institute on Aging, Bethesda, USA; CEO/Consultant Data Tecnica International, Glen Echo, MD, USA), Laurie Robak (Baylor College of Medicine, Houston, Texas, USA), Jose Bras (Center for Neurodegenerative Science, Van Andel Research Institute, Grand Rapids, Michigan, USA), Rita Guerreiro (Center for Neurodegenerative Science, Van Andel Research Institute, Grand Rapids, Michigan, USA), Steven Lubbe (Ken and Ruth Davee Department of Neurology, Northwestern University Feinberg School of Medicine, Chicago, IL, USA), Steven Finkbeiner (Departments of Neurology and Physiology, University of California, San Francisco; Gladstone Institute of Neurological Disease; Taube/Koret Center for Neurodegenerative Disease Research, San Francisco, CA, USA),  Niccolo E. Mencacci (Northwestern University Feinberg School of Medicine, Chicago, IL, USA), Codrin Lungu (National Institutes of Health Division of Clinical Research, NINDS, National Institutes of Health, Bethesda, MD, USA), Andrew B Singleton (Laboratory of Neurogenetics, National Institute on Aging, Bethesda, MD, USA), Sonja W. Scholz (Neurodegenerative Diseases Research Unit, National Institute of Neurological Disorders and Stroke, Bethesda, MD, USA), Xylena Reed (Laboratory of Neurogenetics, National Institute on Aging, Bethesda, MD, USA). Roy N. Alcalay (Department of Neurology, College of Physicians and Surgeons, Columbia University Medical Center, New York, NY, USA, Taub Institute for Research on Alzheimer's Disease and the Aging Brain, College of Physicians and Surgeons, Columbia University Medical Center, New York, NY, USA)

**Canada:** Ziv Gan-Or (Montreal Neurological Institute and Hospital, Department of Neurology & Neurosurgery, Department of Human Genetics, McGill University, Montréal, QC, H3A 0G4, Canada), Guy A. Rouleau (Montreal Neurological Institute and Hospital, Department of Neurology & Neurosurgery, Department of Human Genetics, McGill University, Montréal, QC, H3A 0G4, Canada), Lynne Krohn (Montreal Neurological Institute and Hospital, Department of Neurology & Neurosurgery, Department of Human Genetics, McGill University, Montréal, QC, H3A 0G4, Canada), Lynne Krohn (Montreal Neurological Institute and Hospital, Department of Neurology & Neurosurgery, Department of Human Genetics, McGill University, Montréal, QC, H3A 0G4, Canada),

**The Netherlands:** Jacobus J van Hilten (Department of Neurology, Leiden University Medical Center, Leiden, Netherlands), Johan Marinus (Department of Neurology, Leiden University Medical Center, Leiden, Netherlands)

**Spain:** Astrid D. Adarmes-Gómez (Instituto de Biomedicina de Sevilla (IBiS), Hospital Universitario Virgen del Rocío/CSIC/Universidad de Sevilla, Seville), Miquel Aguilar (Fundació Docència i Recerca Mútua de Terrassa and Movement Disorders Unit, Department of Neurology, University Hospital Mutua de Terrassa, Terrassa, Barcelona.), Ignacio Alvarez (Fundació Docència i Recerca Mútua de Terrassa and Movement Disorders Unit, Department of Neurology, University Hospital Mutua de Terrassa, Terrassa, Barcelona.),Victoria Alvarez (Hospital Universitario Central de Asturias, Oviedo), Francisco Javier Barrero (Hospital Universitario San Cecilio de Granada, Universidad de Granada), Jesús Alberto Bergareche Yarza (Instituto de Investigación Sanitaria Biodonostia, San Sebastián), Inmaculada Bernal-Bernal (Instituto de Biomedicina de Sevilla (IBiS), Hospital Universitario Virgen del Rocío/CSIC/Universidad de Sevilla, Seville), Marta Blazquez (Hospital Universitario Central de Asturias, Oviedo), Marta Bonilla-Toribio (Instituto de Biomedicina de Sevilla (IBiS), Hospital Universitario Virgen del Rocío/CSIC/Universidad de Sevilla, Seville), Juan A. Botía (Universidad de Murcia, Murcia), María Teresa Boungiorno (Fundació Docència i Recerca Mútua de Terrassa and Movement Disorders Unit, Department of Neurology, University Hospital Mutua de Terrassa, Terrassa, Barcelona.) Dolores Buiza-Rueda (Instituto de Biomedicina de Sevilla (IBiS), Hospital Universitario Virgen del Rocío/CSIC/Universidad de Sevilla, Seville), Ana Cámara (Hospital Clinic de Barcelona), Fátima Carrillo (Instituto de Biomedicina de Sevilla (IBiS), Hospital Universitario Virgen del Rocío/CSIC/Universidad de Sevilla, Seville), Mario Carrión-Claro (Instituto de Biomedicina de Sevilla (IBiS), Hospital Universitario Virgen del Rocío/CSIC/Universidad de Sevilla, Seville), Debora Cerdan (Hospital General de Segovia, Segovia), Jordi Clarimón (Memory Unit, Department of Neurology, IIB Sant Pau, Hospital de la Santa Creu i Sant Pau, Universitat Autònoma de Barcelona and Centro de Investigación Biomédica en Red en Enfermedades Neurodegenerativas (CIBERNED), Madrid),Yaroslau Compta (Hospital Clinic de Barcelona), Monica Diez-Fairen (Fundació Docència i Recerca Mútua de Terrassa and Movement Disorders Unit, Department of Neurology, University Hospital Mutua de Terrassa, Terrassa, Barcelona.), Oriol Dols-Icardo (Memory Unit, Department of Neurology, IIB Sant Pau, Hospital de la Santa Creu i Sant Pau, Universitat Autònoma de Barcelona, Barcelona, and Centro de Investigación Biomédica en Red en Enfermedades Neurodegenerativas (CIBERNED), Madrid), Jacinto Duarte (Hospital General de Segovia, Segovia), Raquel Duran (Centro de Investigacion Biomedica, Universidad de Granada, Granada), Francisco Escamilla-Sevilla (Hospital Universitario Virgen de las Nieves, Instituto de Investigación Biosanitaria de Granada, Granada), Mario Ezquerra (Hospital Clinic de Barcelona), Cici Feliz (Departmento de Neurologia, Instituto de Investigación Sanitaria Fundación Jiménez Díaz, Madrid, Spain), Manel Fernández (Hospital Clinic de Barcelona), Rubén Fernández-Santiago (Hospital Clinic de Barcelona), Ciara Garcia (Hospital Universitario Central de Asturias, Oviedo), Pedro García-Ruiz (Instituto de Investigación Sanitaria Fundación Jiménez Díaz, Madrid), Pilar Gómez-Garre (Instituto de Biomedicina de Sevilla (IBiS), Hospital Universitario Virgen del Rocío/CSIC/Universidad de Sevilla, Seville), Maria Jose Gomez Heredia (Hospital Universitario Virgen de la Victoria, Malaga), Isabel Gonzalez-Aramburu (Hospital Universitario Marqués de Valdecilla-IDIVAL, Santander) ,Ana Gorostidi Pagola (Instituto de Investigación Sanitaria Biodonostia, San Sebastián), Janet Hoenicka (Institut de Recerca Sant Joan de Déu, Barcelona), Jon Infante (Hospital Universitario Marqués de Valdecilla-IDIVAL and University of Cantabria, Santander, and Centro de Investigación Biomédica en Red en Enfermedades Neurodegenerativas (CIBERNED), Silvia Jesús (Instituto de Biomedicina de Sevilla (IBiS), Hospital Universitario Virgen del Rocío/CSIC/Universidad de Sevilla, Seville), Adriano Jimenez-Escrig (Hospital Universitario Ramón y Cajal, Madrid), Jaime Kulisevsky (Movement Disorders Unit, Department of Neurology, IIB Sant Pau, Hospital de la Santa Creu i Sant Pau, Universitat Autònoma de Barcelona, Barcelona, and Centro de Investigación Biomédica en Red en Enfermedades Neurodegenerativas (CIBERNED)), Miguel A. Labrador-Espinosa (Instituto de Biomedicina de Sevilla (IBiS), Hospital Universitario Virgen del Rocío/CSIC/Universidad de Sevilla, Seville),  Jose Luis Lopez-Sendon (Hospital Universitario Ramón y Cajal, Madrid), Adolfo López de Munain Arregui (Instituto de Investigación Sanitaria Biodonostia, San Sebastián), Daniel Macias (Instituto de Biomedicina de Sevilla (IBiS), Hospital Universitario Virgen del Rocío/CSIC/Universidad de Sevilla, Seville), Irene Martínez Torres (Department of Neurology, Instituto de Investigación Sanitaria La Fe, Hospital Universitario y Politécnico La Fe, Valencia), Juan Marín (Movement Disorders Unit, Department of Neurology, IIB Sant Pau, Hospital de la Santa Creu i Sant Pau, Universitat Autònoma de Barcelona, Barcelona, and Centro de Investigación Biomédica en Red en Enfermedades Neurodegenerativas (CIBERNED)), Maria Jose Marti (Hospital Clinic Barcelona), Juan Carlos Martínez-Castrillo (Instituto Ramón y Cajal de Investigación Sanitaria, Hospital Universitario Ramón y Cajal, Madrid), Carlota Méndez-del-Barrio (Instituto de Biomedicina de Sevilla (IBiS), Hospital Universitario Virgen del Rocío/CSIC/Universidad de Sevilla, Seville), Manuel Menéndez González (Hospital Universitario Central de Asturias, Oviedo), Marina Mata (Department of Neurology, Hospital Universitario Infanta Sofía, Madrid, Spain) Adolfo Mínguez (Hospital Universitario Virgen de las Nieves, Granada, Instituto de Investigación Biosanitaria de Granada), Pablo Mir (Instituto de Biomedicina de Sevilla (IBiS), Hospital Universitario Virgen del Rocío/CSIC/Universidad de Sevilla, Seville), Elisabet Mondragon Rezola (Instituto de Investigación Sanitaria Biodonostia, San Sebastián), Esteban Muñoz (Hospital Clinic Barcelona), Javier Pagonabarraga (Movement Disorders Unit, Department of Neurology, IIB Sant Pau, Hospital de la Santa Creu i Sant Pau, Universitat Autònoma de Barcelona, Barcelona, and Centro de Investigación Biomédica en Red en Enfermedades Neurodegenerativas (CIBERNED)), Pau Pastor (Fundació Docència i Recerca Mútua de Terrassa and Movement Disorders Unit, Department of Neurology, University Hospital Mutua de Terrassa, Terrassa, Barcelona.), Francisco Perez Errazquin (Hospital Universitario Virgen de la Victoria, Malaga), Teresa Periñán-Tocino (Instituto de Biomedicina de Sevilla (IBiS), Hospital Universitario Virgen del Rocío/CSIC/Universidad de Sevilla, Seville), Javier Ruiz-Martínez (Hospital Universitario Donostia, Instituto de Investigación Sanitaria Biodonostia, San Sebastián), Clara Ruz (Centro de Investigacion Biomedica, Universidad de Granada, Granada), Antonio Sanchez Rodriguez (Hospital Universitario Marqués de Valdecilla-IDIVAL, Santander), María Sierra (Hospital Universitario Marqués de Valdecilla-IDIVAL, Santander), Esther Suarez-Sanmartin (Hospital Universitario Central de Asturias, Oviedo), Cesar Tabernero (Hospital General de Segovia, Segovia), Juan Pablo Tartari (Fundació Docència i Recerca Mútua de Terrassa and Movement Disorders Unit, Department of Neurology, University Hospital Mutua de Terrassa, Terrassa, Barcelona), Cristina Tejera-Parrado (Instituto de Biomedicina de Sevilla (IBiS), Hospital Universitario Virgen del Rocío/CSIC/Universidad de Sevilla, Seville), Eduard Tolosa  (Hospital Clinic Barcelona), Francesc Valldeoriola (Hospital Clinic Barcelona), Laura Vargas-González (Instituto de Biomedicina de Sevilla (IBiS), Hospital Universitario Virgen del Rocío/CSIC/Universidad de Sevilla, Seville), Lydia Vela (Department of Neurology, Hospital Universitario Fundación Alcorcón, Madrid), Francisco Vives (Centro de Investigacion Biomedica, Universidad de Granada, Granada).

**Austria:** Alexander Zimprich (Department of Neurology, Medical University of Vienna, Austria)

**Norway:** Lasse Pihlstrom (Department of Neurology, Oslo University Hospital, Oslo, Norway), Mathias Toft (Department of Neurology and Institute of Clinical Medicine, Oslo University Hospital, Oslo, Norway)

**Estonia**: Sulev Koks (Department of Pathophysiology, University of Tartu, Tartu, Estonia; Department of Reproductive Biology, Estonian University of Life Sciences, Tartu, Estonia), Pille Taba (Department of Neurology and Neurosurgery, University of Tartu, Tartu, Estonia)

**Israel:** Sharon Hassin-Baer (The Movement Disorders Institute, Department of Neurology and Sagol Neuroscience Center, Chaim Sheba Medical Center, Tel-Hashomer, 5262101, Ramat Gan, Israel, Sackler Faculty of Medicine, Tel Aviv University, Tel Aviv, Israel)

**Finland:** Kari Majamaa (Institute of Clinical Medicine, Department of Neurology, University of Oulu, Oulu, Finland; Department of Neurology and Medical Research Center, Oulu University Hospital, Oulu, Finland), Ari Siitonen (Institute of Clinical Medicine, Department of Neurology, University of Oulu, Oulu, Finland; Department of Neurology and Medical Research Center, Oulu University Hospital, Oulu, Finland)

**Nigeria:** Njideka U. Okubadejo (University of Lagos, Lagos State, Nigeria), Oluwadamilola O. Ojo (University of Lagos, Lagos State, Nigeria),

**Kazakhstan:** Coordinator - Rauan Kaiyrzhanov **(**Department of Molecular Neuroscience, UCL Institute of Neurology, London, UK), Chingiz Shashkin (Kazakh National Medical University named after Asfendiyarov, Almaty, Kazakhstan), Nazira Zharkynbekova (South Kazakhstan Medical Academy, Shymkent, Kazakhstan), Vadim Akhmetzhanov (Astana Medical University, Astana Kazakhstan), Akbota Aitkulova (National Center for Biotechnology, Astana, Kazakhstan; Al-Farabi Kazakh national university. Almaty Kazakhstan), Elena Zholdybayeva (National Center for Biotechnology, Astana, Kazakhstan), Zharkyn Zharmukhanov (National Center for Biotechnology, Astana, Kazakhstan), Gulnaz Kaishybayeva (Scientific and practical center “Institute of neurology named after Smagul Kaishybayev”, Almaty, Kazakhstan), Altynay Karimova (Scientific and practical center “Institute of neurology named after Smagul Kaishybayev”, Almaty, Kazakhstan), Dinara Sadykova (Astana Medical University, Astana, Kazakhstan).
